# GDF15 is required for maintaining subcutaneous adipose tissue lipid metabolic signature

**DOI:** 10.1038/s41598-024-77448-w

**Published:** 2024-11-06

**Authors:** Carla Igual-Gil, Christopher A. Bishop, Markus Jähnert, Kornelia Johann, Verena Coleman, Vanessa Baum, Michael Kruse, Andreas F.H. Pfeiffer, Olga Pivovarova-Ramich, Mario Ost, Maximilian Kleinert, Susanne Klaus

**Affiliations:** 1 Department of Physiology of Energy Metabolism, German Institute of Human Nutrition Potsdam-Rehbrücke, Arthur-Scheunert-Allee 114-116, 14458 Nuthetal, Germany; 2 Institute of Nutritional Science, University of Potsdam, Arthur-Scheunert-Allee 114-116, 14458 Nuthetal, Germany; 3 Department of Experimental Diabetology, German Institute of Human Nutrition Potsdam-Rehbrücke, Arthur-Scheunert-Allee 114-116, 14458 Nuthetal, Germany; 4 Department of Molecular Physiology of Exercise and Nutrition, German Institute of Human Nutrition Potsdam-Rehbrücke, Arthur-Scheunert-Allee 114-116, 14458 Nuthetal, Germany; 5 Paul Flechsig Institute of Neuropathology, University Clinic Leipzig, Leipzig, Germany; 6 German Center for Diabetes Research (DZD e.V.), Neuherberg, Germany; 7 Department of Clinical Nutrition, German Institute of Human Nutrition Potsdam-Rehbruecke, Nuthetal, Germany; 8 Department of Endocrinology, Diabetes and Nutrition, Charité – Universitätsmedizin Berlin, Corporate Member of Freie Universität Berlin, Humboldt-Universität zu Berlin, Campus Benjamin Franklin, Berlin, Germany; 9 Department of Endocrinology and Metabolism, Charité – Universitätsmedizin Berlin, Corporate Member of Freie Universität Berlin, Humboldt-Universität zu Berlin, Berlin, Germany; 10 Department of Molecular Metabolism and Precision Nutrition, German Institute of Human Nutrition Potsdam-Rehbruecke, Nuthetal, Germany

**Keywords:** Fat metabolism, Homeostasis, Metabolic diseases

## Abstract

Recent research has identified growth differentiation factor 15 (GDF15) as a crucial factor in various physiological and pathological processes, particularly in energy balance regulation. While the role of GDF15 in modulating energy metabolism through hindbrain GDNF family receptor alpha-like (GFRAL) signaling has been extensively studied, emerging evidence suggests direct peripheral metabolic actions of GDF15. Using knockout mouse models, we investigated GDF15 and GFRAL’s roles in adipose tissue metabolism. Our findings indicate that C57BL/6/129/SvJ Gdf15-KO mice exhibit impaired expression of *de novo* lipogenesis enzymes in subcutaneous adipose tissue (sWAT). In contrast, C57BL/6J Gfral-KO mice showed no impairments compared to wild-type (WT) littermates. RNA-Seq analysis of sWAT in Gdf15-KO mice revealed a broad downregulation of genes involved in lipid metabolism. Importantly, our study uncovered sex-specific effects, with females being more affected by GDF15 loss than males. Additionally, we observed a fasting-induced upregulation of GDF15 gene expression in sWAT of both mice and humans, reinforcing this factor’s role in adipose tissue lipid metabolism. In conclusion, our research highlights an essential role for GDF15 in sWAT lipid metabolic homeostasis. These insights enhance our understanding of GDF15’s functions in adipose tissue physiology and underscore its potential as a therapeutic target for metabolic disorders.

**Supplementary Information:**

The online version contains supplementary material available at 10.1038/s41598-024-77448-w.

## Introduction

In the last years, the role of growth differentiation factor 15 (GDF15) in health and disease has been extensively studied. GDF15 was first identified in 1997 in activated macrophages and described as a divergent member of the transforming growth factor β (TGFβ) superfamily^1,2^. Since its discovery, different actions have been attributed to GDF15 such as having a cardioprotective role upon myocardial infarction^3^, or being involved in the cancer inflammatory response^4^. In recent years, however, interest on GDF15 has increased since the identification of its receptor GDNF family receptor alpha-like (GFRAL), expressed exclusively in the area postrema (AP) and nucleus of the solitary tract (NTS) in the hindbrain of rodents. The identification of GFRAL allowed the discovery of one of the most described functions of GDF15: the suppression of food intake via hindbrain signaling^5–8^. Since then, a large body of literature has proven that, when GDF15 is induced upon different kinds of physiological and pathological stressors, such as mitochondrial dysfunction^9^ as well as upon ingestion of some dietary components such as fatty acids^10,11^, it reduces food intake in rodents via GFRAL receptor signaling. Additionally, GDF15-GFRAL signaling has been shown to be involved in other pathophysiological processes such as nausea, gastric emptying^12,13^, or leading to anxiety-like behaviors^14^. Remarkably, in a recent study, GDF15-GFRAL signaling was shown to be able to promote weight loss by inducing energy expenditure in muscle^15^. Furthermore, it has been reported that GDF15-GFRAL signaling increases insulin action in the liver and adipose tissue via β-adrenergic signaling^16^. Along these lines, in a mouse model of cancer cachexia, GDF15 through GFRAL signaling proved to induce lipolysis of WAT through activation of sympathetic tone^17^. These strong effects of the GDF15-GFRAL axis in the control of energy balance have encouraged attempts to develop clinically relevant GDF15 receptor agonists for the potential treatment of metabolic diseases^18^. Nevertheless, despite the remarkable and well-reported role of GDF15 through GFRAL signaling, there is emerging evidence to suggest that GDF15 might also act directly on peripheral metabolic tissues, in a GFRAL-independent manner. These advances, however, have been in some cases hampered by the use of mammalian-derived recombinant GDF15 contaminated with TGF-β^19^ and as such, interpretation of data generated using these compounds should be done with care. The liver, being one of the tissues with highest GDF15 expression in mice, has been one of the most studied regarding local metabolic actions of this factor. In a study aiming to understand the progression of non-alcoholic hepatic steatohepatitis (NASH), it was found that β-Arrestin 1 (ARRB1) played a key protective role through facilitating maturation of GDF15^20^. Furthermore, it was recently shown that GDF15 activates AMP-activated protein kinase (AMPK), in turn inhibiting gluconeogenesis and fibrosis by modulating the activity of the TGF-β1/SMAD3 pathway^21^. A direct metabolic action of GDF15 in other peripheral tissues has been less studied. In skeletal muscle, GDF15 has been reported to also activate AMPK, thereby mediating the effects of peroxisome proliferator-activated receptor β/δ (PPARβ/δ), such as increasing fatty acid oxidation, in a GFRAL-independent manner^22^. In brown adipose tissue (BAT), GDF15 has been proposed to be involved in the thermogenic response, but whether it acts independently of GFRAL signaling still remains unclear^23,24^. While also limited, there is evidence that could reflect GFRAL-independent actions of GDF15 in white adipose tissue (WAT). On one hand, GDF15 overexpression in mice led to body weight as well as WAT loss without changes in food intake^23^. Furthermore, in cultured human adipocytes, skeletal muscle-derived GDF15 led to increases in basal lipolysis^25^.

Given the limited data regarding the role of GDF15 in the control of adipose tissue metabolism in vivo, we here aimed to characterize and further explore the lipid metabolic phenotype of *ad libitum* fed male and female Gdf15-knockout (Gdf15-KO) mice using wildtype (WT) littermates as controls, with a special focus on adipose tissue depots. Hereby, we find that GDF15 plays a crucial role in maintaining lipid metabolic signature specifically of subcutaneous (sWAT), but not of gonadal adipose tissue (gWAT). Interestingly, we found no differences in the sWAT lipid metabolic signature of Gfral-KO mice compared to their WT littermates, indicating that GFRAL signaling does not play a major role in the modulation of lipid metabolism under fed conditions. Furthermore, we reveal sex-specific effects, showing that loss of GDF15 has stronger effects on the sWAT transcriptional lipid metabolic profile of females compared to male mice. Finally, we show that GDF15 is induced in sWAT of both mice and humans during fasting, further reinforcing a role for GDF15 in the control of lipid metabolism in this tissue.

## Results

### GDF15 tissue expression and Gdf15-KO mice phenotypic characterization

**Fig. 1:**
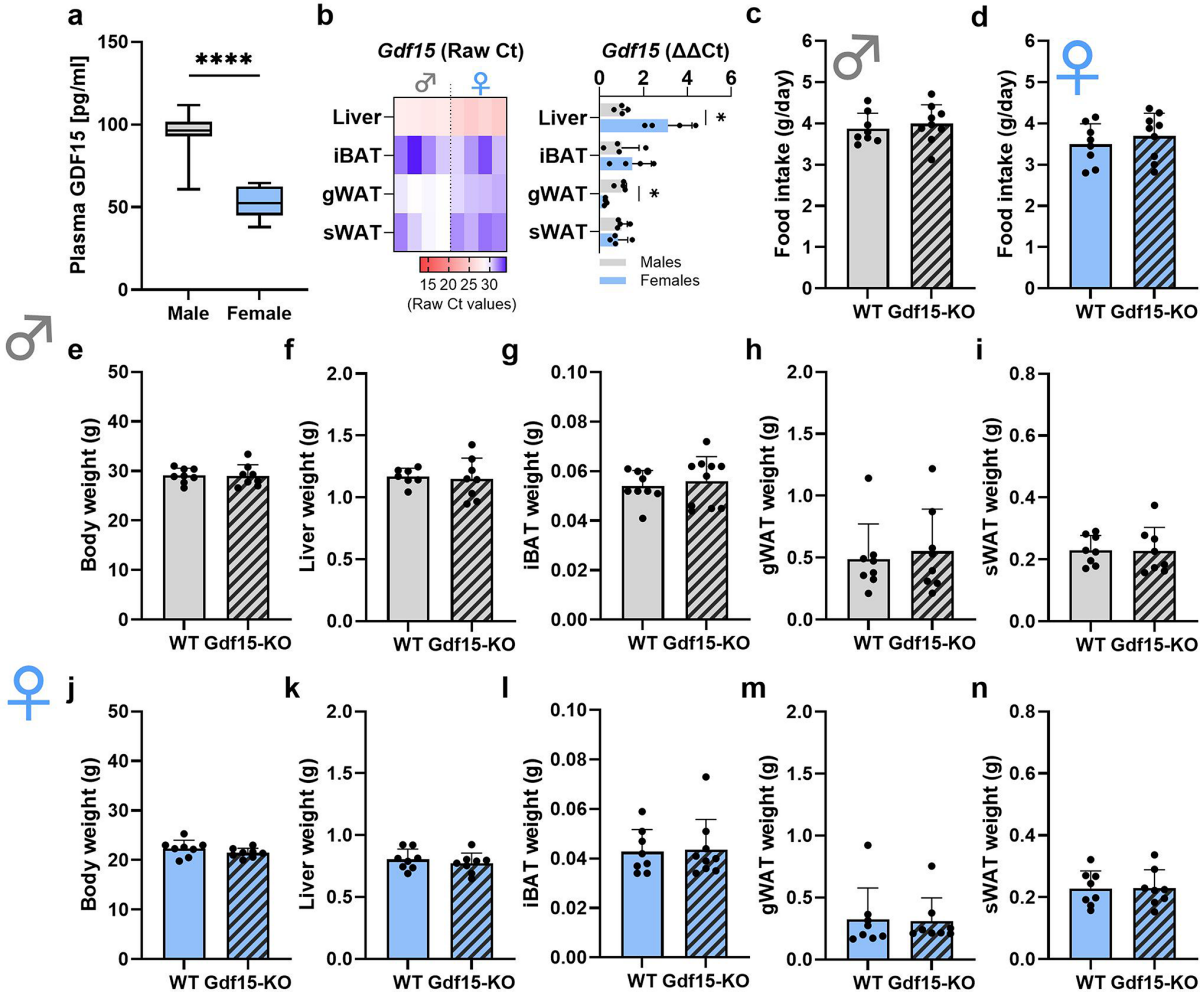
Loss of GDF15 does not affect body and metabolic tissue weights. (**a**) Circulating GDF15 levels and (**b**) *Gdf15* gene expression (expressed in raw Ct values (left) or relative to male ΔΔCt (right)) in liver, interscapular brown adipose tissue (iBAT), gonadal adipose tissue (gWAT) and subcutaneous adipose tissue (sWAT) in male and female WT mice. (**c**) 24-hour food intake in male and (**d**) female WT and Gdf15-KO mice. (**e**,** j**) Body weight, (**f**,** k**) liver weight, (**g**,** l**) iBAT weight, (**h**,** m**) gWAT weight and (**i**,** n**) sWAT weight in WT and Gdf15-KO, male and female mice, respectively. Data are presented as mean ± SD with single data points. *P < 0.05; **P < 0.01; ***P < 0.001; ****P < 0.0001. Statistical analyses were performed using an unpaired t-test. WT: wildtype; Gdf15-KO: Gdf15 knockout; Age: 20 weeks.

First, we aimed to characterize basal levels of circulating GDF15 and *Gdf15* gene expression of the main tissues involved in lipid metabolism in wildtype (WT) male and female mice, respectively (Fig. 1a, b). Of note, WT male mice exhibited plasma GDF15 concentrations of around 100 pg/ml, while WT female mice showed lower GDF15 circulating levels of about 50 pg/ml (Fig. 1a). *Gdf15* gene expression in both male and female mice was highest in the liver, followed by gonadal adipose tissue (gWAT), subcutaneous adipose tissue (sWAT) and brown adipose tissue (iBAT), respectively. Regarding sex differences, liver *Gdf15* expression was higher in females than in males, whereas it was higher in gWAT of males compared to females (Fig. 1b).

In order to investigate the effects of the loss of GDF15 in the main adipose tissue depots of mice in a sex-specific manner, we made use of whole-body Gdf15-KO mice and performed a careful metabolic and molecular phenotyping. Importantly, mice were sacrificed in the fed state in order to avoid fasting-induced GDF15 effects^26^. Both male and female Gdf15-KO mice exhibited no changes in food intake compared to their WT littermates (Fig. 1c, d). Furthermore, we observed no changes in body (Fig. 1e, j) or tissue weights (liver, sWAT, gWAT, and iBAT) (Fig. 1f-i, k-n) of Gdf15-KO mice compared to WT, independent of sex.

### Effect of the loss of GDF15 in adipose tissue depots

Morphological examination of the liver with histological analyses revealed no visible differences between WT and Gdf15-KO mice (Fig. S1a, i). This was in line with the analysis of liver triglycerides (TG) and glycogen (Fig. S1b, c, j, k), which was unaffected in both male and female Gdf15-KO mice compared to WT mice. qPCR analysis of players in the main molecular pathways involved in the liver’s metabolic actions further revealed no major effects of the loss of GDF15 (Fig. S1d-h, l-p), indicating that GDF15 does not play a major role in liver metabolic regulation and lipid metabolic pathways under fed conditions.

**Fig. 2:**
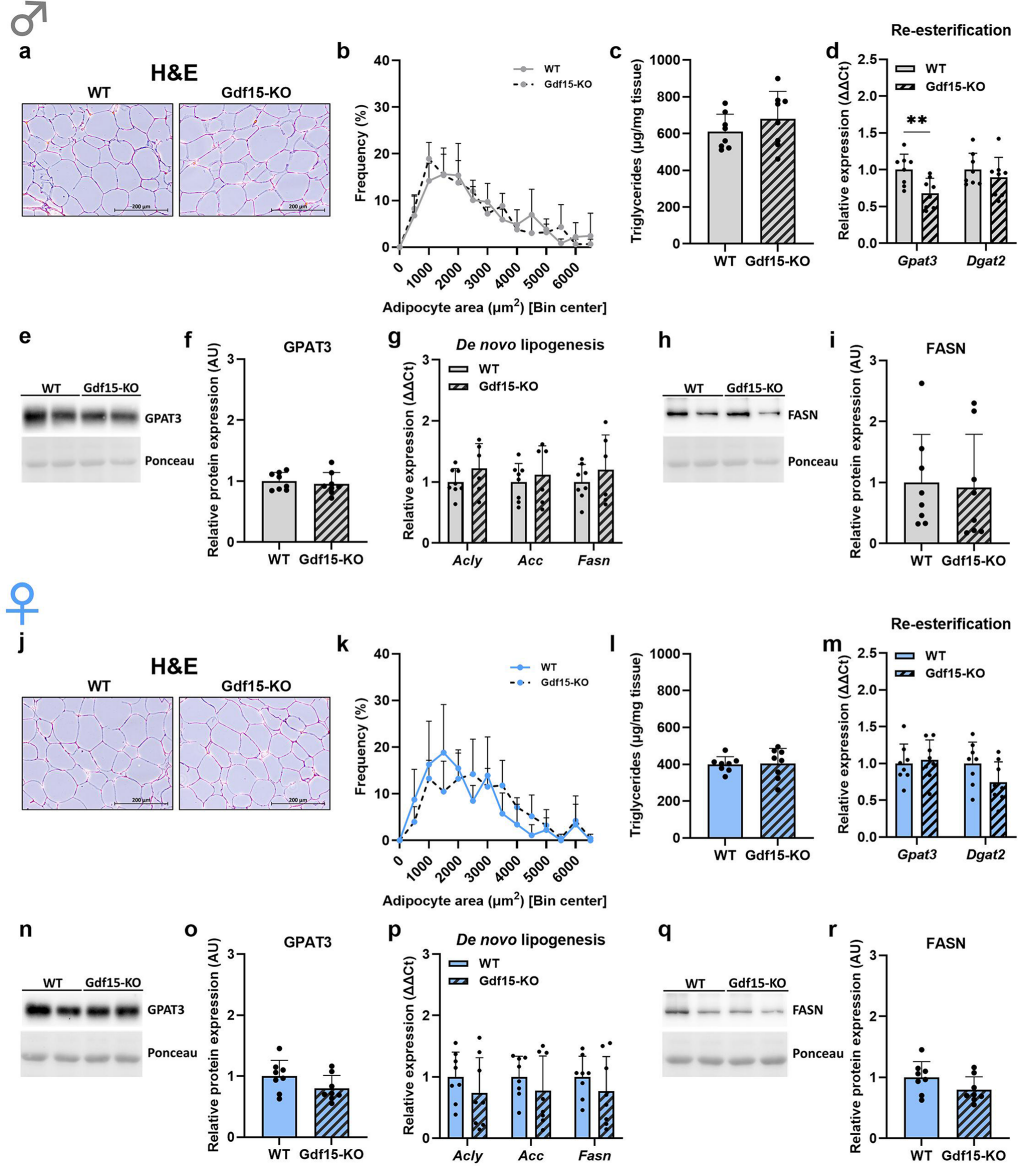
Loss of GDF15 does not affect the lipid metabolic profile of gonadal adipose tissue. (**a**,** j**) Representative H&E staining of gonadal adipose tissue (gWAT) and (**b**,** k**) adipocyte area frequency. (**c**,** l**) gWAT triglyceride content. (**d**,** m**) gWAT relative gene expression of re-esterification enzymes *Gpat3* and *Dgat2* and (**e**,** n**) western blot analysis of GPAT3 protein expression (representative cropped blot) and (**f**,** o**) quantification. (**g**,** p**) gWAT relative gene expression of *de novo* lipogenesis enzymes *Acly*, *Acc* and *Fasn* and (**h**,** q**) western blot analysis of FASN protein expression (representative cropped blot) and (**i**,** r**) quantification. Data are presented as mean ± SD with single data points (c-i, l-r). *P < 0.05; **P < 0.01; ***P < 0.001; ****P < 0.0001. Statistical analyses were performed using an unpaired t-test. WT: wildtype; Gdf15-KO: Gdf15 knockout. Age: 20 weeks.

We next sought to address the lipid metabolic signature of adipose tissue depots in Gdf15-KO mice. gWAT H&E staining (Fig. 2a, j) and adipocyte area quantification (Fig. 2b, k) revealed no morphological differences in adipocyte size distribution between Gdf15-KO and WT mice, independent of sex. This was further supported by the fact that loss of GDF15 did not affect TG content of gWAT in either male or female mice (Fig. 2c, l). Nonetheless, we performed further molecular analyses of re-esterification as well as *de novo* lipogenesis pathways in order to get a closer look into lipid metabolic processes that might be affected by the loss of GDF15. Gene expression of the re-esterification enzyme glycerol-3-phosphate acyltransferase 3 (*Gpat3*) was significantly reduced in male Gdf15-KO mice compared to WT, while diacylglycerol O-acyltransferase 2 *(Dgat2)* expression was not affected by the loss of GDF15 (Fig. 2d). In female Gdf15-KO mice, gene expression of these enzymes was unchanged compared to their WT littermates (Fig. 2m). In order to further validate these findings, we performed western blot analyses of GPAT3, which revealed no differences in the expression of this enzyme at the protein level in either male or female mice (Fig. 2e, f, n, o). Furthermore, gene expression analyses of *de novo* lipogenesis enzymes ATP citrate lyase *(Acly)*, acetyl-CoA-carboxylase *(Acc)* and fatty acid synthase *(Fasn)* revealed no changes between WT and Gdf15-KO male or female mice (Fig. 2g, p), which was further confirmed on the protein level for FASN (Fig. 2h, i, q, r). Altogether, these data indicate that loss of GDF15 does not affect the regulation of lipid metabolism of gWAT in either male or female mice.

**Fig. 3:**
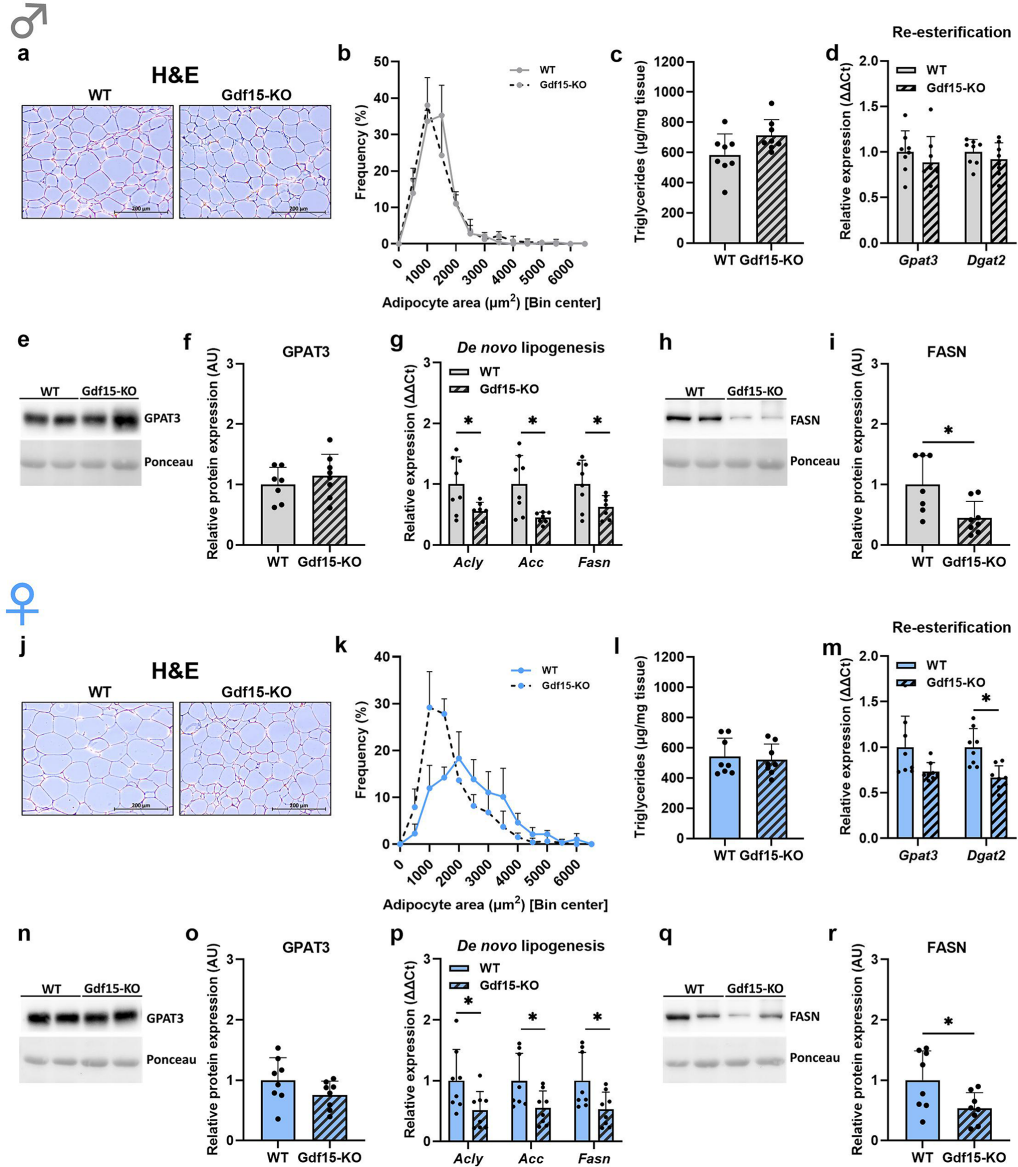
GDF15 modulates lipid metabolism of subcutaneous adipose tissue. (**a**,** j**) Representative H&E staining of subcutaneous adipose tissue (sWAT) and (**b**,** k**) adipocyte area frequency. (**c**,** l**) sWAT triglyceride content. (**d**,** m**) sWAT relative gene expression of re-esterification enzymes *Gpat3* and *Dgat2* and (**e**,** n**) western blot analysis of GPAT3 protein expression (representative cropped blot) and (**f**,** o**) quantification. (**g**,** p**) sWAT relative gene expression of *de novo* lipogenesis enzymes *Acly*, *Acc* and *Fasn* and (**h**,** q**) western blot analysis of FASN protein expression (representative cropped blot) and (**i**,** r**) quantification. Data are presented as mean ± SD with single data points (c-i, l-r). *P < 0.05; **P < 0.01; ***P < 0.001; ****P < 0.0001. Statistical analyses were performed using an unpaired t-test. WT: wildtype; Gdf15-KO: Gdf15 knockout. Age: 20 weeks.

Next, we aimed to elucidate whether GDF15 plays a role in the regulation of lipid metabolism of sWAT (Fig. 3). Interestingly, while morphological observation and adipocyte area quantification revealed no differences between Gdf15-KO and WT male mice (Fig. 3a, b), female Gdf15-KO mice appeared to have a different distribution of adipocyte area compared to WT mice, namely a higher frequency of smaller adipocytes in sWAT (Fig. 3j, k). We further analyzed TG content of sWAT, which was unchanged in either male or female Gdf15-KO mice compared to their WT littermates (Fig. 3c, l). Gene expression of re-esterification enzymes *Gpat3* and *Dgat2* was not affected by the loss of GDF15 in either male or female mice (Fig. 2d, m), which was in line with the protein levels of GPAT3 (Fig. 3e, f, n, o). Strikingly, in both male and female mice, expression of the key *de novo* lipogenesis genes, *Acly*, *Acc* and *Fasn*, was significantly reduced in Gdf15-KO mice compared to WT (Fig. 3g, p). These results were further confirmed by the analysis of FASN protein expression, which was also significantly decreased in Gdf15-KO mice compared to WT (Fig. 3h, i, q, r). From these results we conclude that in sWAT, loss of GDF15 affects the expression of *de novo* lipogenesis enzymes and, in female mice, it further induces a reduction in adipocyte size.

**Fig. 4:**
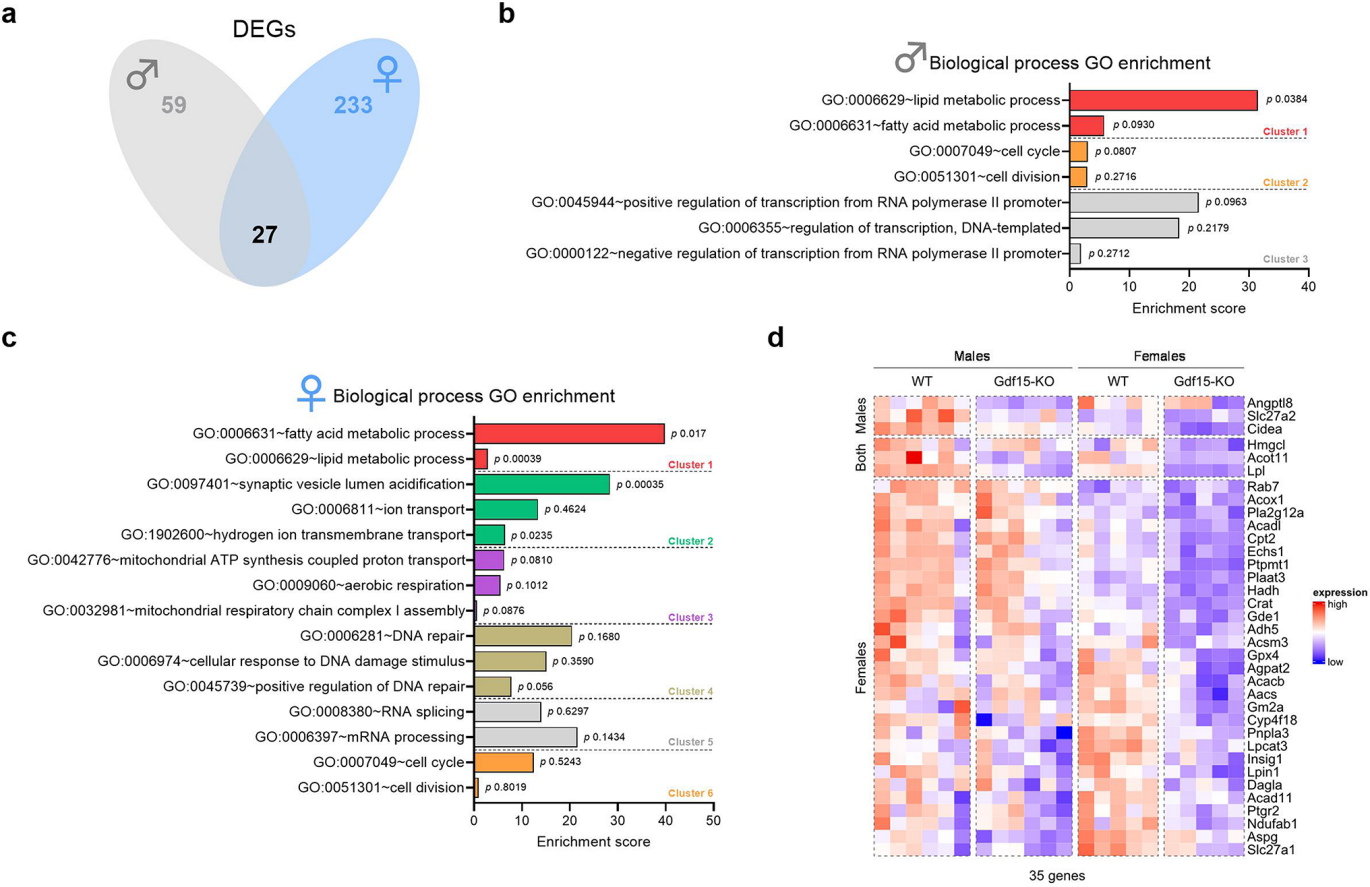
Loss of GDF15 leads to a downregulation of genes allocated to fatty acid and lipid metabolic pathways which is more pronounced in females. (**a**) Overlap between males and females of the total differentially expressed genes (DEGs) between Gdf15-KO and WT mouse subcutaneous adipose tissue (sWAT). (**b**,** c**) Pathway enrichment analysis of DEGs in male and female mice, respectively. (**d**) Heatmap of lipid metabolism-related DEGs that were significant only in males (top), both sexes (middle) and only in females (bottom). WT: wildtype; Gdf15-KO: Gdf15 knockout. Age: 20 weeks.

In order to further explore these findings, we performed RNA-Seq analyses of sWAT tissue in Gdf15-KO male and female mice, with corresponding control WT littermates (Fig. 4). In line with our previous findings, analysis of the total differentially expressed genes (DEGs) between Gdf15-KO and WT mice revealed that females are more affected by the loss of GDF15 than males, with 233 and 59 DEGs respectively, and an overlap of 27 DEGs between the two sexes (Fig. 4a). This was further confirmed by principal component analysis (PCA) (Fig. S2), which indicated a separation between sexes independent of genotype (Fig. S2a) and a greater separation of genotypes among females (Fig. S2b) than among males (Fig. S2c). Pathway analysis revealed, for both male and female mice, that pathways related to lipid metabolism were the most affected by the loss of GDF15 (Fig. 4b, c). Finally, examination of the specific DEGs allocated to lipid metabolism pathways in males, females, and both sexes (Fig. 4d) revealed an overall downregulation of genes involved in fatty acid synthesis, oxidation and mobilization. Of note, sex/genotype interaction analysis by Two-way ANOVA of the expression of these genes revealed no interaction of these two variables (supplementary Table 1). Altogether, these results further strengthen the conclusion that GDF15 plays a crucial role in the regulation of lipid metabolism in sWAT.

### Effect of the loss of GFRAL in subcutaneous adipose tissue

**Fig. 5:**
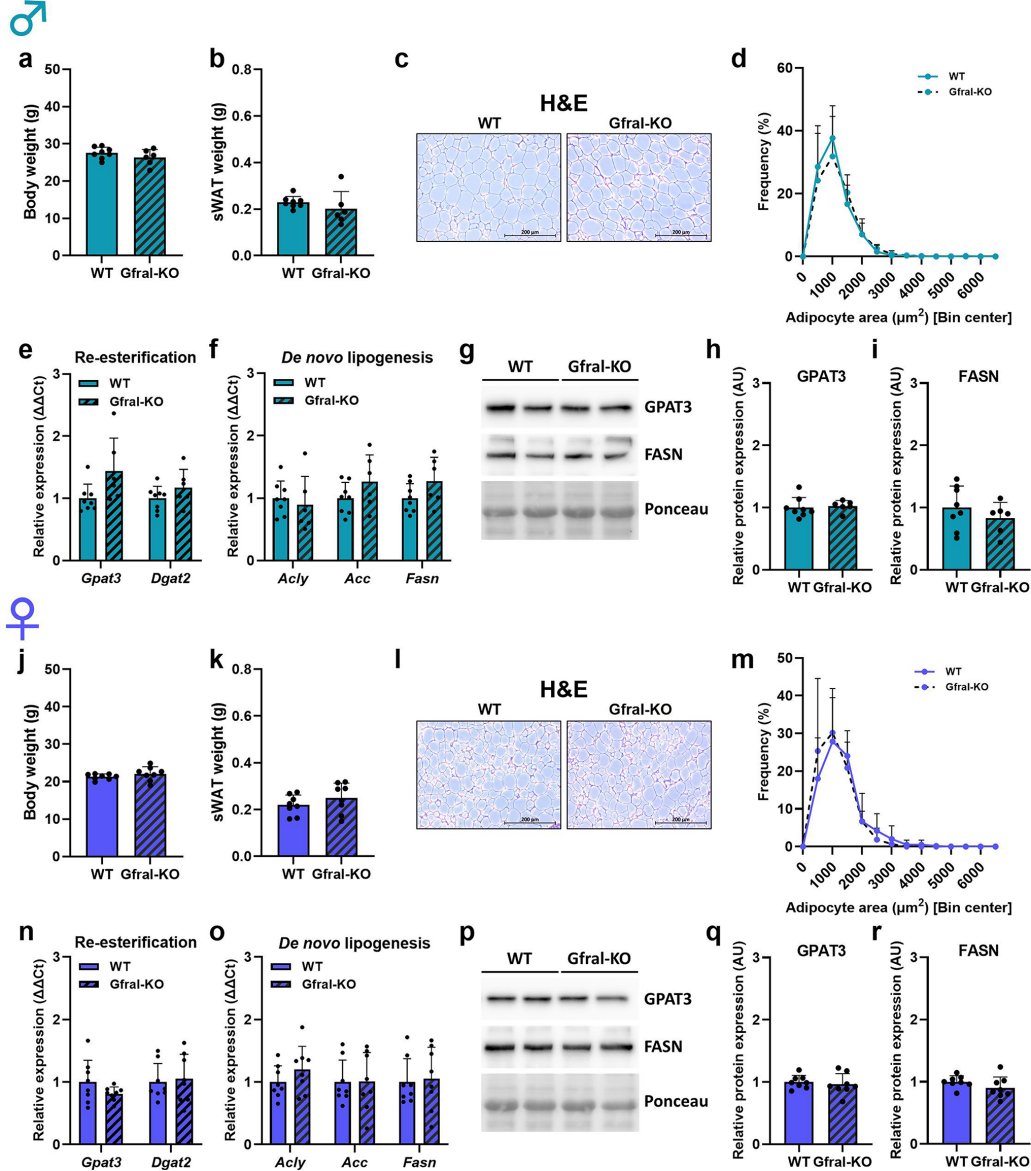
GFRAL signaling does not play a role in GDF15-dependent effects on lipid metabolism in subcutaneous white adipose tissue. (**a**,** j**) Body and (**b**,** k**) subcutaneous adipose tissue (sWAT) in WT and Gfral-KO, male and female mice, respectively. (**c**,** l**) Representative H&E staining of sWAT and (**d**,** m**) adipocyte area frequency. (**e**,** n**) sWAT relative gene expression of re-esterification enzymes *Gpat3* and *Dgat2* and (**f**,** o**) *de novo* lipogenesis enzymes *Acly*, *Acc* and *Fasn* in WT and Gfral-KO, male and female mice, respectively. (**g**,** p**) Western blot analysis of GPAT3 and FASN protein expression (representative cropped blots) and (**h**,** i**,** q**,** r**) respective quantifications in WT and Gfral-KO, male and female mice. Data are presented as mean ± SD with single data points. Statistical analyses were performed using an unpaired t-test. WT: wildtype; Gfral-KO: Gfral knockout. Age: 20 weeks.

In order to determine the involvement of the GFRAL receptor in sWAT lipid metabolism, we performed a metabolic characterization of male and female Gfral-KO mice using WT littermates as controls (Fig. 5). Both body weight (Fig. 5a, j) and sWAT tissue weight (Fig. 5b, k) were unaffected by loss of GFRAL, consistent with previous reports^6^. Importantly, loss of GFRAL did not affect adipocyte morphology and size in either male or female mice (Fig. 5c, d, l, m), which was consistent with no changes in gene expression of both re-esterification enzymes *Gpat3* and *Dgat2* (Fig. 5e, n) and *de novo* lipogenesis enzymes *Acly*, *Acc* and *Fasn* (Fig. 5f, o). These findings were further supported by the analysis of protein expression of GPAT3 and FASN, which was also unaffected in Gfral-KO mice compared to WT (Fig. 5g-i, p-r). Taken together, these data indicate that GFRAL signaling is not involved in the modulation of sWAT lipid metabolism. Due to differences in the genetic background of Gdf15-KO and Gfral-KO mice used in our study we cannot, however, completely exclude a possible GFRAL involvement in the reported effects of GDF15 in sWAT and therefore these results need to be interpreted accordingly.

### Effect of fasting in GDF15 expression in adipose tissue of mice and humans

**Fig. 6:**
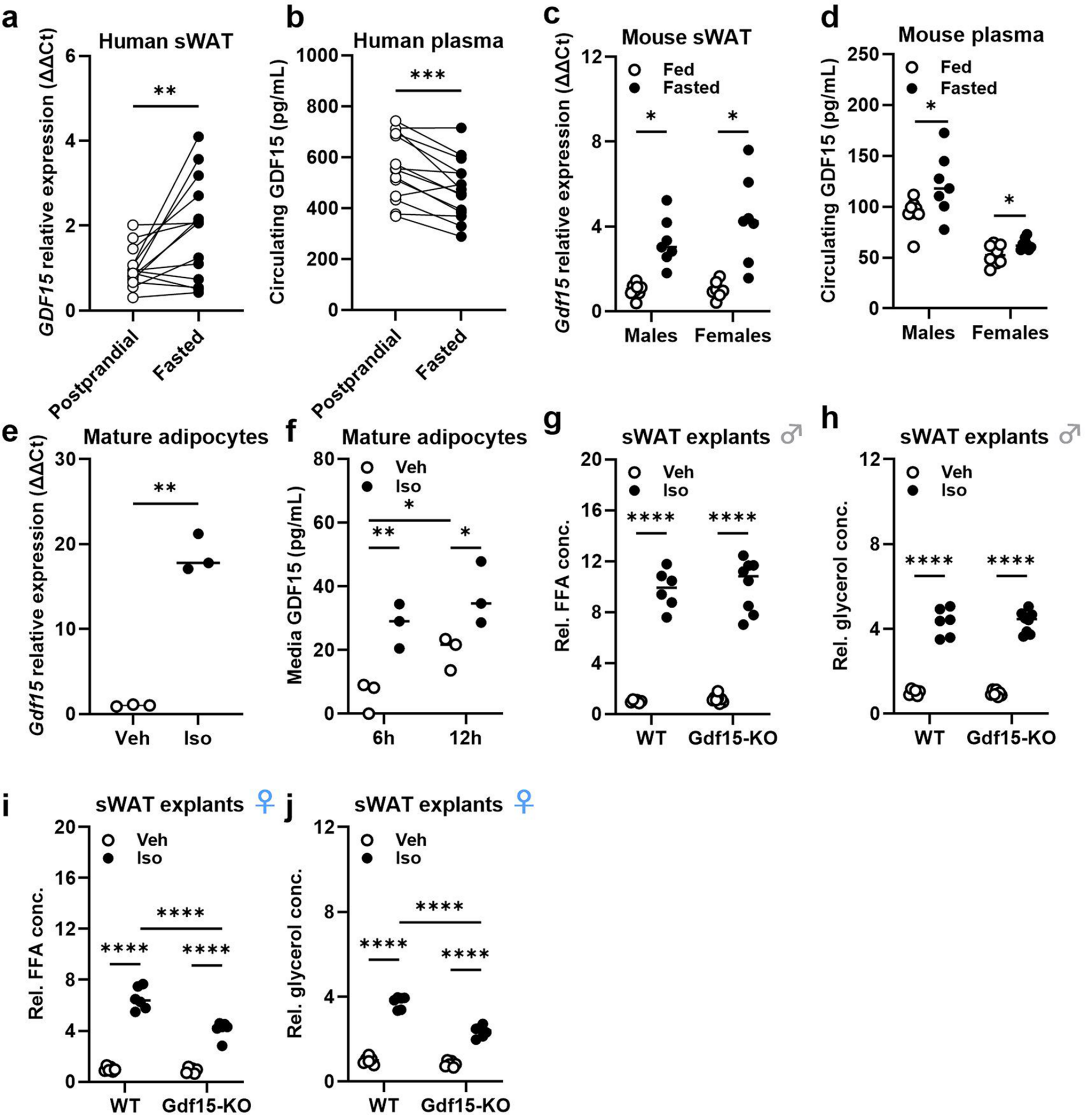
GDF15 is involved in the fasting response of subcutaneous adipose tissue. (**a**) Postprandial and fasted (15 h) *GDF15* gene expression levels in sWAT biopsies and (**b**) plasma GDF15 of moderately obese men. (**c**) *Gdf15* gene expression in sWAT and (**d**) plasma GDF15 of fed and fasted (24 h) 20-week old male and female WT mice. (**d**) *Gdf15* gene expression in membrane mature adipocyte aggregate cultures (MAAC) after a 4 h isoproterenol treatment. (**f**) Media GDF15 levels of MAAC after a 6 h and 12 h isoproterenol stimulation. (**g-j**) Media relative free fatty acids (FFA) and glycerol after an isoproterenol stimulation of sWAT explants of WT and Gdf15-KO male (**g**,** h**) and female (**i**,** j**) mice. Data are presented as single data points. *P < 0.05; **P < 0.01; ***P < 0.001; ****P < 0.0001. Statistical analyses were performed using a paired t-test (a, b), unpaired t-test (c-e) or a Two-way ANOVA (g-j).

As previously mentioned, GDF15 has been shown to be induced in the liver during the fasting response, where it plays a role in promoting fatty acid oxidation and ketogenesis^26^. Considering our previous results and given the fact that adipose tissue plays a crucial role in the fasting response, we aimed to investigate a potential role for GDF15 in fasting of sWAT. In a human cohort of moderately obese men we could show that, after 15 h of fasting, *GDF15* gene expression in sWAT biopsies was significantly induced (Fig. 6a), while circulating GDF15 levels were significantly reduced (Fig. 6b). In mice, a 24-hour fasting challenge significantly induced both sWAT *Gdf15* gene expression (Fig. 6c) and circulating GDF15 (Fig. 6d) in both males and females. Of note, unlike in sWAT, the effect of fasting on *Gdf15* expression in gWAT, iBAT and liver was sex-specific (Fig. S3). To determine whether this fasting-induced increase in sWAT *Gdf15* is specific to adipocytes or originates from other cell types (e.g., macrophages), we isolated primary mature adipocytes from sWAT and treated them with the β-adrenergic agonist isoproterenol, replicating the fasting response in vitro. Thereby, we could confirm that β-adrenergic stimulation strongly induces *Gdf15* gene expression in an adipocyte-specific manner (Fig. 6e). Further, measurement of GDF15 in the supernatant of mature adipocytes treated with isoproterenol for 6 and 12 h showed a significant induction in the media of isoproterenol-treated cells compared to control cells (Fig. 6f), indicating that β-adrenergic stimulation leads to GDF15 secretion by adipocytes. Finally, in order to further understand a potential role for GDF15 in the fasting response of adipose tissue, we treated sWAT explants in vitro of WT and Gdf15-KO mice with isoproterenol and measured free fatty acids (FFA) and glycerol in the supernatant as a readout for lipolysis activation (Fig. 6g-j). Interestingly, while we observed no differences in FFA or glycerol of male sWAT explants between genotypes (Fig. 6g, h), female Gdf15-KO sWAT explants treated with isoproterenol showed reduced FFA and glycerol in the supernatant compared to WT explants (Fig. 6i, j), indicating that loss of GDF15 affects the lipolytic response, at least in female mice.

## Discussion

The role of GDF15 and its only known hindbrain-restricted receptor GFRAL has been extensively studied in the past years due to its relevance in the control of energy homeostasis and, thereby, being a potential therapeutic target in the control of metabolic diseases such as obesity^27^. However, recent investigations point to GFRAL-independent actions of GDF15 in metabolically active tissues such as the liver, adipose tissue or skeletal muscle which suggests that GDF15 may act as a mediator of energy metabolism apart from its effects on food intake^21–23,25^. Here, with the aim to further investigate a potential metabolic role for GDF15 in lipid metabolism, we performed a metabolic characterization of Gdf15-KO male and female mice with a focus on the main adipose tissue depots. With this investigation we show that GDF15 is involved in the regulation of lipid metabolic processes, specifically in sWAT of both male and female mice, with female mice being more affected by the loss of GDF15. Finally, we show that fasting induces an upregulation of GDF15 expression in sWAT of mice and humans, and that adrenergic stimulation of female Gdf15-KO sWAT explants ex vivo leads to an impaired free fatty acid and glycerol release compared to WT explants, indicating that GDF15 might be involved in lipid mobilization or fatty acid oxidation in adipose tissue during this response.

Notably, despite the fact that sWAT is the tissue in which *Gdf15* gene expression is lowest in both sexes among the tissues investigated here, we find a remarkable effect on lipid metabolism in this tissue upon loss of GDF15 in Gdf15-KO mice. Furthermore, while *Gdf15* gene expression presents sex differences in liver and gWAT, it shows similar levels of expression among sexes in sWAT, the tissue where we also find sex differences in lipid metabolism upon loss of GDF15. Due to the limited availability of functioning GDF15 antibodies specifically for western blot, it is difficult to determine the actual intra-tissue levels of GDF15. Therefore, it is hard to draw definite conclusions on protein expression of GDF15 among the different tissues. Nevertheless, we cannot rule out the possibility that the effects of GDF15 loss observed in sWAT, including sex-specific differences, are a secondary effect to the loss of GDF15 in another metabolically relevant tissue. Of note, while both sWAT and gWAT have similar tissue structures and a lipid storage function, we only identify a GDF15 action in the lipid metabolic regulation of sWAT and not gWAT. This could be attributed to the fact that sWAT appears to have a more metabolically active signature as well as a higher storage capacity, while gWAT presents a more inflammatory signature and is linked to the development of metabolic disorders^28,29^. Importantly, subcutaneous adipose tissue, rather than visceral adipose tissue, is considered the primary physiological site for lipid storage in humans^30^, indicating that our findings may be more relevant to human physiology.

Despite the increasing interest in GDF15, its physiological role during the fasting response in humans remains poorly understood. In mice, we observe a modest increase in GDF15 levels of both males and females after a 24 h challenge while previous research reported no difference in GDF15 levels upon fasting^31^. While time of sacrifice was not provided in the cited study, mice were sacrificed at “lights off” in our investigation. Given the circadian nature attributed to GDF15 in humans and under certain stress-conditions in mice^9,32^, time of the day in which experiments were performed could explain these discrepancies in GDF15 induction during fasting. Furthermore, the origin of circulating GDF15 during fasting remains unclear. Interestingly, we here see that there are sex-specific effects on *Gdf15* expression in different metabolic organs upon fasting. While in male mice *Gdf15* appears to be upregulated in sWAT, liver and iBAT during fasting, female mice mainly present an induction of this factor only in adipose tissue depots. When evaluating these effects, however, we need to carefully consider that all mice were sacrificed after 24 h of fasting and therefore, these sex dimorphisms could represent different stages in the fasting response of male and female mice considering their differences in body weight and composition. In humans, previous research has demonstrated that prolonged fasting results in a peak of circulating GDF15 levels after 48 h, followed by a decline over a period of up to 7 days^31^. Further, in overweight or obese women, GDF15 was shown to be increased with intermittent fasting and these increases were not associated with food intake^33^. In our study, a 15 h fast in moderately obese men led to significantly lower GDF15 circulating levels, but also to a significant induction in sWAT GDF15 gene expression, indicating that sWAT might not be contributing to circulating GDF15 during the first stages of fasting in humans. Therefore, GDF15 might play a GFRAL-independent, auto-/paracrine role in sWAT under this physiological response. Our adrenergic stimulation experiments performed in sWAT explants show an impaired lipolytic capacity upon the lack of GDF15, which would suggest an implication of this factor in the lipolytic response. It is important to point out that these experiments were performed using whole-body knockout mice and therefore, the effects observed might represent an indirect effect. In this regard, we cannot rule out a possible neuroendocrine action of GDF15 in vivo during fasting, since GDF15 has been reported to induce cortisol^34^, which could have an influence on adipose tissue physiology. Thus, further research is needed to elucidate the molecular mechanisms behind GDF15’s actions in adipose tissue.

The molecular mechanisms by which GDF15 affects sWAT lipid metabolism cannot be determined from our data. We here show that Gfral-KO mice do not show alterations in their adipose tissue lipid metabolic profile compared to their WT littermates, indicating that GFRAL does not play a role in the modulation of adipose tissue and could point to GFRAL-independent GDF15 effects. It is important to point out, however, that Gfral-KO and Gdf15-KO mice used in this investigation had C57BL/6J and C57BL/6/129/SvJ genetic backgrounds, respectively, and since genetic background could have an effect on phenotypic outcomes, interpretation of our results should be done with care. It has been hypothesized that GDF15 might act on peripheral tissues through other so-far unidentified receptors^35^. As we show here, the lipolytic response to adrenergic stimulation was impaired in female Gdf15-KO sWAT, which further reinforces a direct, GFRAL-independent role for GDF15 in modulating lipid metabolism. We cannot rule out, however, that these effects are due to adaptations to the lack of GDF15 in Gdf15-KO mice. Furthermore, the use of mammalian-derived recombinant GDF15 contaminated with TGF-β^19^ in some studies makes it hard to draw conclusions on GDF15 mechanisms of action, and therefore, we are still far from understanding them. GDF15 has been shown to be present in the nucleus and to be implicated in direct transcriptional regulation of the Smad pathway^36^, and thus, it is possible that GDF15 exerts a transcriptional control of lipid metabolic genes in sWAT. Furthermore, we cannot completely rule out the possibility that the effects observed on sWAT in vivo are due to GDF15 originating from other tissues such as liver or skeletal muscle, which would suggest that GDF15 may have an alternative endocrine mode of action independent of GFRAL signaling.

Altogether, our data indicate that loss of GDF15 in sWAT of Gdf15-KO mice leads to a lipid metabolic remodeling that includes the downregulation of both catabolic (e.g. lipolysis and lipid oxidation) but also anabolic (e.g. *de novo* lipogenesis) pathways, which contrasts with the catabolic roles attributed in other tissues such as liver or skeletal muscle^22^. It should be noted that here we used knockout mice which are adapted to the loss of GDF15 since birth. Thus, the fact that the amount and TG content of sWAT was not affected in GDF15-KO mice despite their apparently decreased *de novo* lipid synthesis capacity suggests that this downregulation of anabolic enzymes could represent a compensatory mechanism. This hypothesis is further strengthened by the reported fasting-induced sWAT GDF15 upregulation, which indicates that GDF15 might play a supportive role in lipolytic and/or oxidative pathways during this energy demanding state.

Our research points out sex-specific effects of GDF15 loss in the remodeling of sWAT lipid metabolism. Interestingly, while males presented both higher sWAT *Gdf15* mRNA expression and circulating levels, our data reveals that female Gdf15-KO mice appear to have a stronger sWAT remodeling compared to male mice. While GDF15 has been suggested to cause nausea and vomiting during pregnancy in women^37^, most studies regarding GDF15’s metabolic actions have been performed in male mice. There is, however, some evidence showing sex-specific GDF15 actions with regards to lipid metabolism. In mice ubiquitously expressing human GDF15 (hNAG-1 mice), a decreased WAT weight with increased expression of WAT lipolytic genes was observed despite no changes in food intake^23^. Interestingly, female NAG-1 mice lost a higher percentage in total WAT mass compared to their WT littermates than male mice did, which, in line with our results, indicate that female mice are more affected by GDF15 metabolic actions in WAT. On the contrary, however, while Gdf15-KO male mice are more prone to body weight gain when fed a high fat diet than WT mice, female Gdf15-KO mice seem to be partially protected from this effect^38^, which could be explained by the fact that female mice are less sensitive to high fat diet-induced obesity than male mice. Moving forward, more studies that specifically address sex differences will be necessary in order to understand GDF15’s implications in metabolic regulation in a sex-specific manner.

Finally, unlike most research on GDF15 focusing on its role as a stress-induced factor in different pathological and pathophysiological conditions, our study highlights its relevance in maintaining tissue homeostasis in unchallenged conditions. Thus, future research using Gdf15-KO mice will need to consider potential tissue-specific metabolic alterations in these mice when being used as unchallenged controls or for pharmacological studies.

## Methods

### Animal studies

Animal experiments were designed and performed in accordance with the relevant guidelines and regulations. For all the procedures of the study we followed the ARRIVE guidelines. Animal studies were approved by the ethics committee of the Ministry of Agriculture and Environment (State Brandenburg, Germany, file numbers GZ 2347-9‐2016 and AZ 2347-14-2021). C57BL/6/129/SvJ whole body Gdf15-knockout (Gdf15-KO) mice were kindly provided by Dr. Se-Jin Lee (University of Connecticut School of Medicine, Department of Genetics and Genome Sciences)^2^. Gfral-heterozygous mice were purchased from Mutant Mouse Regional Resource Centers (MMRRC) and back-crossed to a C57BL/6J background using speed congenics. All mice used in the study were bread in-house by crossing heterozygous mice. Offspring was genotyped and wildtype (WT) and knockout (Gdf15-KO or Gfral-KO) littermates were used for the experiments. Mice were group‐housed and random‐caged with *ad libitum* access to a standard chow diet (Sniff, Soest, Germany) and water at 23 °C and a 12:12‐h dark‐light cycle. At 20 weeks of age, *ad libitum* fed animals were euthanized at “lights off” by an overdose of isoflurane followed by a final cardiac puncture and tissues were dissected, snap-frozen and kept at -80 °C until further processing. For fasting experiments (Fig. 6, Fig. S3), food was removed 24 h prior to sacrifice in the fasted group, while the fed group kept *ad libitum* access to food. Sacrifice at “lights off”. i.e. in the early activity phase of mice was performed in order to match the early morning of humans, which is the timepoint in which human studies are commonly performed.

### Human study

Human samples analyzed in this study are a subset obtained from a larger study conducted by our collaborators^39^. Specifically, we examined plasma and subcutaneous adipose tissue biopsies from 14 moderately obese men, all collected at 1:00 p.m. either after an overnight fast (fasting started at 10:00 p.m. the previous day, fasted group) or following a test meal consumed at 9:00 a.m. (postprandial group). The study protocol was conducted in accordance with all relevant guidelines and regulations, was approved by the Ethics Committee of the University of Potsdam (1/2010) and registered with Current Controlled Trials (http://www.controlled-trials.com), ISRCTN22073289. Informed consent was obtained from all participants.

### Mature adipocyte cultures and treatment

Mouse mature adipocytes were isolated from sWAT and cultured following the membrane mature adipocyte aggregate cultures (MAAC) method developed by Harms and colleagues^40^. 24 h after isolation, MAAC were treated either with isoproterenol (1 µM) or vehicle (water) for 4 h, 6–12 h. Cells were harvested with TRIzol for further RNA isolation and media was collected.

### Subcutaneous adipose tissue explant cultures

Subcutaneous adipose tissue (sWAT) of 70-week-old WT and Gdf15-KO, male and female mice was dissected and cut into 60 mg explants. sWAT explants were placed in 600 uL of DMEM F-12 with 10% fatty acid-free BSA and were treated either with isoproterenol (1 µM) or vehicle (water) for 4 h, after which media was collected and free fatty acids (FFA) and glycerol was measured. For the measurement of FFA and glycerol, we used the commercially available kits #NEFA-HR(2) (Wako) and #MAK117 (Sigma-Aldrich), respectively, according to manufacturer’s instructions.

### Plasma GDF15 analyses

Mouse and human plasma GDF15 as well as cell culture media GDF15 concentration were measured with the R&D ELISA Kits #MGD150 and #DGD150, respectively, following manufacturer instructions.

### Gene expression analyses

Unless otherwise stated, RNA was isolated following a TRIzol (Thermo Fisher, #15596026)-chloroform extraction and DNase digested following manufacturer instructions (Fisher Scientific, #EN0521). RNA from MAAC samples as well as human sWAT samples was isolated using a RNeasy Mini Kit (Qiagen, #74104). Complementary DNA was synthesized using the RT SuperMix Kit (NEB, LunaScript, #E3010) and further diluted to a final concentration of 5ng/µL. qPCR analyses were performed in 384 well plates (VWR International GmbH, #731 − 0193) and a Quantstudio 7 device (Fisher Scientific) was used for measurement. Per reaction, 1 µL of cDNA (5 ng/µL) and 4 µL of a master mix containing 2.5 µL of Luna Universal Master Mix (NEB, #M3003E), 0,5 µL of 3 µM forward and reverse primers and 0,5 µL of DEPC water was used. Primer sequences used are the following: *Acat1*: 5´ AGCACACTGAACGATGGAG 3´ (F), 5´ CGCAAGTGGAAAATCAATGGG 3´ (R), *Acc*: 5´ TTTCACTGTGGCTTCTCCAG 3´ (F), 5´ TGCATTTCACTGCTGCAATA 3´ (R), *Acly*: 5´ TATGCCAAGACCATCCTCTCACT 3´(F), 5´TCTCACAATGCCCTTGAAGGT 3´ (R), *Acox1*: 5´ CACCATTGCCATTCGATACA 3´ (F), 5´ TGCGTCTGAAAATCCAAAATC 3´ (R), *Aqp9*: 5´ GGAAGGATGGAGTGGTTCAA 3´ (F), 5´ GGCCATGAGTCCGTCATAGT 3´ (R); *B2m*: 5′ CCCCACTGAGACTGATACATACGC 3′ (F), 5′ AGAAACTGGATTTGTAATTAAGCAGGTTC3′ (R), *Bdh1*: 5′ TGCAACAGTGAAGAGGTGGAGAAG 3′ (F), 5′ CAAACGTTGAGATGCCTGCGTTGT3′ (R), *Cd36*: 5′ CCAAGCTATTGCGACATGAT 3′ (F), 5′ ACAGCGTAGATAGACCTGCAAA 3′ (R), *Cpt1a*: 5′ CCAAACCCACCAGGCTACA 3′ (F), 5′ GCACTGCTTAGGGATGTCTCTATG 3′ (R), *Dgat2*: 5´ TGCTAGGAGTGGCCTGCAGTGT3´ (F), 5´CACTGCGATCTCCTCCCACCTT 3´ (R), *Fabp1*: 5′ TCACCATCACCTATGGACCCA 3′ (F), 5′ GCTTGACGACTGCCTTGACTTT 3′ (R), *Fasn*: 5′ TTGATGATTCAGGGAGTGGA 3′ (F), 5′ TTACACCTTGCTCCTTGCTG 3′ (R), *Gpat1*: 5′ CTTGGCCGATGTAAACACAC 3′ (F), 5′ TCCTTCCATTTCAGTGTTGC 3′ (R), *Gpat3*: 5′ TCTCCTCCGAAGAGCTAGTATCATG 3′ (F), 5′ ACCCAGCACCCACACCAT 3′ (R), *Hmgcl*: 5′ ACTACCCAGTCCTGACTCCAA 3′ (F), 5′ TAGAGCAGTTCGCGTTCTTCC 3′ (R), *Hmgcs2*: 5′ GAAGAGAGCGATGCAGGAAAC 3′ (F), 5′ GTCCACATATTGGGCTGGAAA 3′ (R), *Gdf15*: 5′ GAGCTACGGGGTCGCTTC 3′ (F), 5′ GGGACCCCAATCTCACCT 3′ (R); (human) *RPLP0*: 5’ GCTTCCTGGAGGGTGTCC 3’ (F), 5’ GGACTCGTTTGTACCCGTTG 3’ (R); (human) *GDF15*: 5’ GCTTCCTGGAGGGTGTCC 3’ (F), 5’ GGACTCGTTTGTACCCGTTG 3’ (R).

### Histological analyses

Tissues were dissected and fixed in 4% formaldehyde for 24 h, after which they were embedded in paraffin and cut into 2 μm slices (HM 355 S Rotatory Microtom). Slices were mounted, dehydrated with increasing ethanol concentrations, and stained with hematoxylin and eosin (H&E) (Roth, Fluka). Quantification of adipocyte area was performed using the Adiposoft (Fiji plug-in) software (v1.16; https://imagej.net/plugins/adiposoft).

### Triglyceride measurement

Tissue triglycerides were measured using a triglyceride reagent kit (Sigma Aldrich, #T2449-10ML) after extraction with either hypotonic lysis buffer (10 mmol/L sodium phosphate buffer (pH 7.4) containing 1 mmol/L EDTA and 1% polyoxyethylene (10) tridecyl ether) for liver samples or 15% IGEPAL buffer (Merck, #56741) for adipose tissue samples.

### Glycogen measurement

Liver glycogen content was measured using a Starch Analysis Kit (R-Biopharm AG, #10207748035) following manufacturer instructions after extraction with 1 N NaOH.

### Western blot analysis

Tissues were homogenized in RIPA buffer containing Halt Protease/Phosphatase Inhibitor Cocktail (Thermo Fisher, #78440) and protein concentration was determined using the DC Protein Assay Reagent (Biorad, #500-0114). 15 µg of protein were loaded on an SDS gel. The primary antibodies used were GPAT3 (CST, #96164) and FASN (CST, #3189S). An anti-rabbit horseradish peroxidase‐conjugated secondary antibody was used (#7074, Cell Signaling Technology). Densitometry was determined using ImageJ (https://imagej.net/ij/) and values were normalized to the respective WT control group.

### RNA sequencing

The RNA sequencing was carried out by BGI Genomics laboratory in Hong Kong via nanoball sequencing technology. Raw 150 bp paired reads were adapter trimmed and quality controlled. The filtered sequences resulted in ~ 9GB reads per sample. BGI aligned the reads to the GRCm39 reference using Bowtie2 (v2.2.5; https://bowtie-bio.sourceforge.net/bowtie2/index.shtml) and gene expression was calculated via RSEM (v1.2.8; https://deweylab.github.io/pRSEM/). Differential gene expression was performed via DESeq2 (v1.38.3; https://bioconductor.org/packages/release/bioc/html/DESeq2.html) pipeline. For pathway enrichment analysis, we used the DAVID Functional Annotation Tool (https://david.ncifcrf.gov/) from the National Institutes of Health (NIH) clustering genes with the annotation categories UP_KW_BIOLOGICAL_PROCESS and GOTERM_BP_DIRECT. The heatmap in Fig. 4 was created via R(v4.1.2) package ComplexHeatmap (v2.10.0; https://www.rdocumentation.org/packages/ComplexHeatmap/versions/).

### Statistical analysis

Statistical analyses were performed using GraphPad Prism version 10.0.0, GraphPad Software, Boston, Massachusetts, USA, www.graphpad.com. Data were tested for normality using a Shapiro-Wilk test, and statistical differences were determined with either an unpaired t-test (mouse data) or a paired t-test (human data). For ex vivo experiments as well as media GDF15 measurements depicted in Fig. 6, a two-way ANOVA with Tukey’s multiple comparisons test was used. Data are presented as single data points or as mean ± SD with single data points. *P < 0.05; **P < 0.01; ***P < 0.001; ****P < 0.0001.

## Electronic supplementary material

Below is the link to the electronic supplementary material.

Supplementary Material 1

## Data Availability

All data supporting these findings are available upon request. The RNA-Seq dataset generated and analyzed in this study is available in the GEO repository (https://www.ncbi.nlm.nih.gov/geo/), accession number GSE272291.
