# Supplementary material for: GDF15 is required for maintaining subcutaneous adipose tissue lipid metabolic signature
Source: Sci Rep. 2024 Nov 6;14:26989. doi: 10.1038/s41598-024-77448-w (PMC11541726; doi:10.1038/s41598-024-77448-w)

Supplementary information

GDF15 is required for maintaining subcutaneous adipose tissue lipid metabolic signature in a GFRAL-independent manner

Carla Igual-Gil[1], Christopher Allen Bishop[1], Markus Jähnert[3,6], Kornelia Johann[4], Verena Coleman[1], Vanessa Baum[2], Michael Kruse[7,8], Andreas F.H. Pfeiffer[6,7,8], Olga Pivovarova-Ramich[6,7,9,10], Mario Ost[1,5], Maximilian Kleinert[4], Susanne Klaus[1,2, *]

[1]Department of Physiology of Energy Metabolism, German Institute of Human Nutrition Potsdam-Rehbrücke, Arthur-Scheunert-Allee 114-116, 14458 Nuthetal, Germany

[2]University of Potsdam, Institute of Nutritional Science, Arthur-Scheunert-Allee 114-116, 14458 Nuthetal, Germany

[3]Department of Experimental Diabetology, German Institute of Human Nutrition Potsdam-Rehbrücke, Arthur-Scheunert-Allee 114-116, 14458 Nuthetal, Germany

[4]Department of Molecular Physiology of Exercise and Nutrition, German Institute of Human Nutrition Potsdam-Rehbrücke, Arthur-Scheunert-Allee 114-116, 14458 Nuthetal, Germany

[5]Paul Flechsig Institute of Neuropathology, University Clinic Leipzig, Leipzig, Germany

[6]German Center for Diabetes Research (DZD e.V.), Neuherberg, Germany

[7]Department of Clinical Nutrition, German Institute of Human Nutrition Potsdam-Rehbruecke, Nuthetal, Germany

[8]Charité – Universitätsmedizin Berlin, Corporate Member of Freie Universität Berlin, and Humboldt-Universität zu Berlin, Department of Endocrinology, Diabetes and Nutrition, Campus Benjamin Franklin, Berlin, Germany;

[9]Charité – Universitätsmedizin Berlin, Corporate Member of Freie Universität Berlin, and Humboldt-Universität zu Berlin, Department of Endocrinology and Metabolism, Berlin, Germany

[10]Department of Molecular Metabolism and Precision Nutrition, German Institute of Human Nutrition Potsdam-Rehbruecke, Nuthetal, Germany

*Correspondence: klaus@dife.de

Figure S1

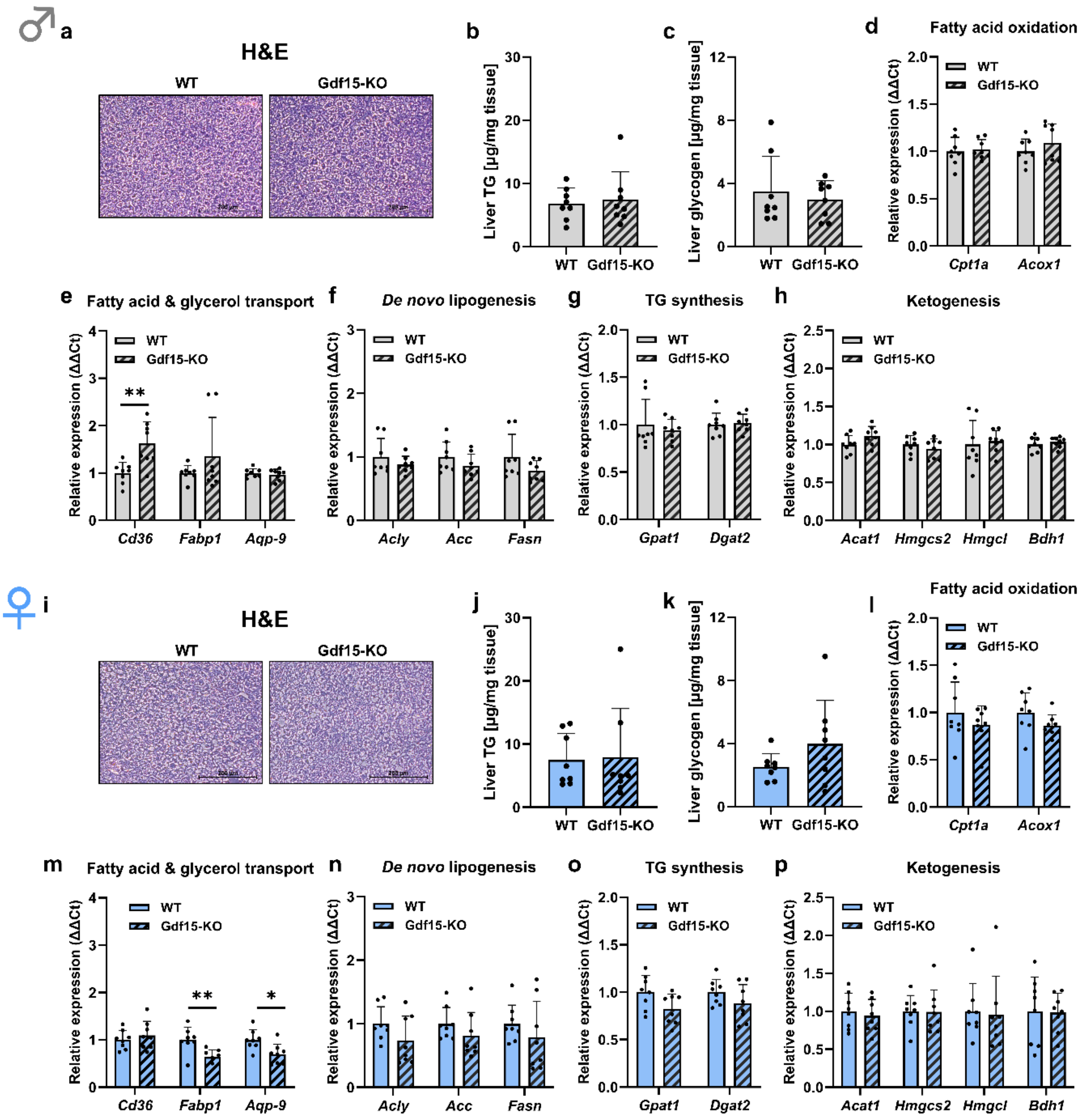

Figure S1. GDF15 does not play a role in liver fat metabolism under fed conditions. (**a, i**) Representative liver H&E staining, (**b, j**) liver triglyceride and (**c, k**) glycogen content, (**d, l**) relative gene expression of fatty acid oxidation enzymes *Cpt1a* and *Acox1*, (**e, m**) relative gene expression of fatty acid and glycerol transport genes *Cd36*, *Fapt1* and *Aqp-9*, (**f, n**) relative gene expression of *de novo* lipogenesis enzymes *Acly*, *Acc* and *Fasn,* (**g, o**) relative gene expression of TG synthesis enzymes *Gpat1* and *Dgat2* and (**h, p**) relative gene expression of ketogenesis enzymes *Acat1*, *Hmgcs2*, *Hmgcl* and *Bdh1* in WT and Gdf15-KO, male and female mice, respectively. Data are presented as mean ± SD with single data points. *P < 0.05; **P < 0.01; ***P < 0.001; ****P < 0.0001. Statistical analyses were performed using an unpaired t-test. WT: wildtype; Gdf15-KO: Gdf15 knockout. Age: 20 weeks.

Figure S2

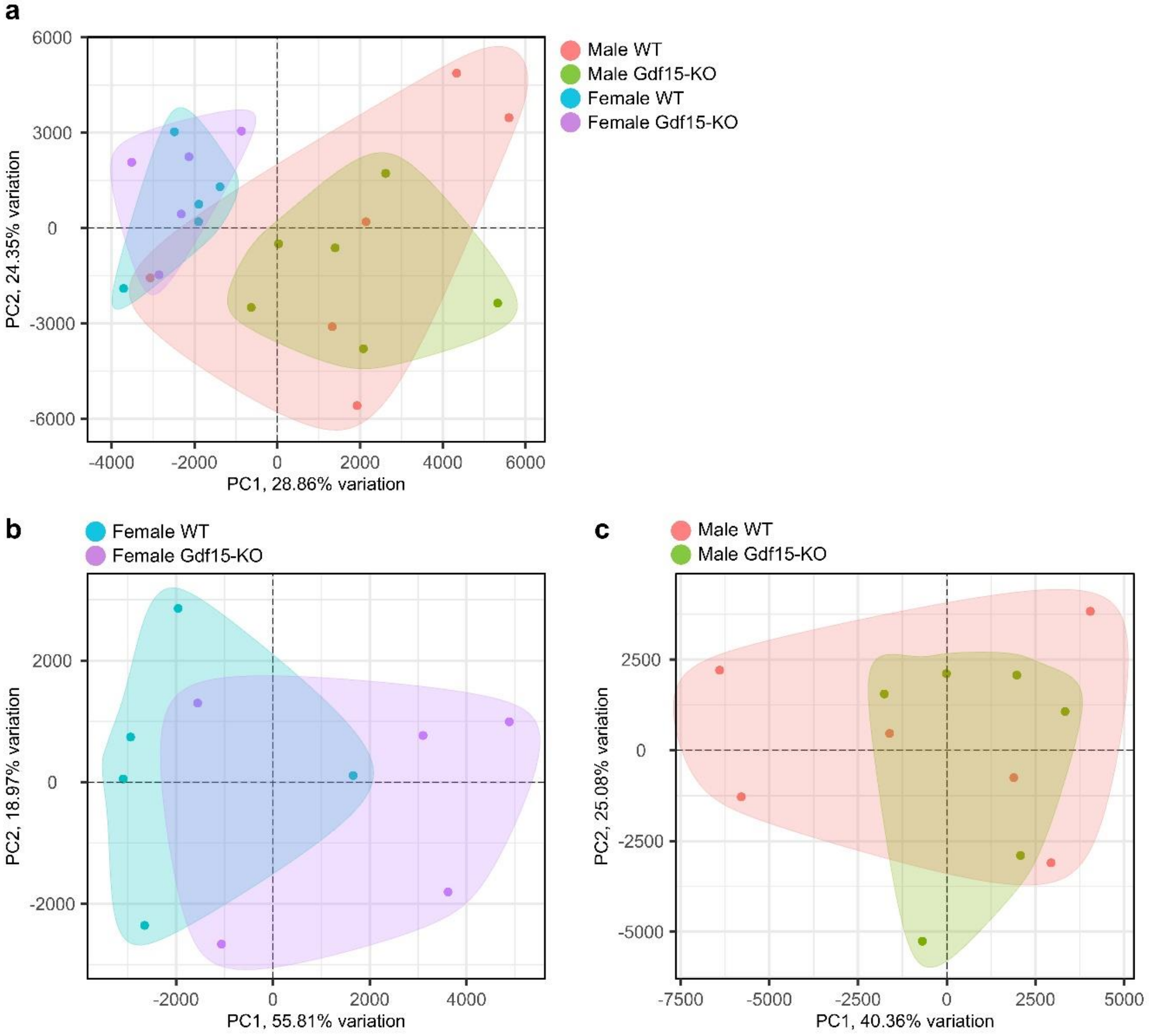

Figure S2. Principal component analysis (PCA) of RNA-Seq results presented in Figure 4. (**a**) PCA of all samples. (**b**) PCA of female samples. (**c**) PCA of male samples. WT: wildtype; Gdf15-KO: Gdf15 knockout.

Figure S3

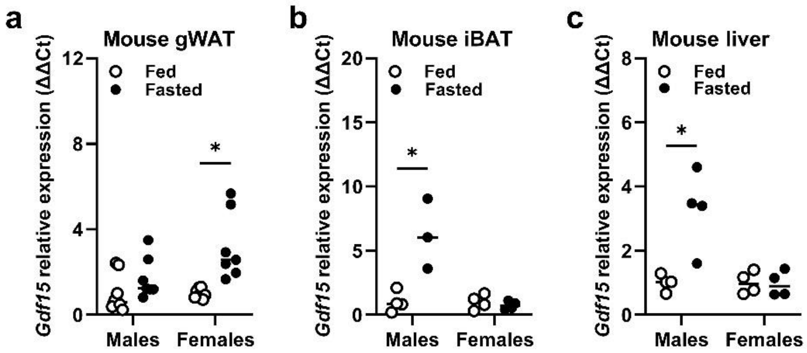

Figure S3. Fasting induces GDF15 expression in a tissue and sex-specific manner. Relative *Gdf15* gene expression of fed and fasted (24 h) male and female mice in (**a**) gonadal adipose tissue (gWAT), (**b**) interscapular brown adipose tissue (iBAT) and (**c**) liver. Data are presented as single data points. *$P < 0.05$; **$P < 0.01$; ***$P < 0.001$; ****$P < 0.0001$. Statistical analyses were performed using an unpaired t-test. Age: 20 weeks.

Table 1

Gene	*p* interaction	*p* sex	*p* genotype
Angptl8	0.5375	0.1436	0.0792
Slc27a2	0.054	**0.0009**	**0.0076**
Cidea	0.3205	**<0.0001**	**0.0003**
Lpl	0.4174	**<0.0001**	**<0.0001**
Acot11	0.3291	0.2133	**0.0018**
Hmgcl	0.202	**0.0092**	0.0545
Acsm3	0.9332	0.5122	**0.037**
Ptgr2	0.4473	0.8603	**0.0421**
Ndufab1	0.1826	0.8361	**0.029**
Acox1	0.767	**0.0002**	0.2184
Cpt2	0.2278	**0.0002**	0.2044
Aacs	0.1121	0.292	**0.0067**
Pnpla3	0.3853	0.1827	**0.0023**
Acad11	0.5289	0.1304	**0.0154**
Aspg	0.1582	**0.0001**	**0.0005**
Gm2a	**0.0245**	0.9302	**0.0304**
Crat	0.5168	**0.0001**	**0.0245**
Rab7	0.7688	**<0.0001**	0.2822
Dagla	0.4903	0.9618	**0.0216**
Adh5	0.2748	**0.0184**	0.1453
Insig1	0.1441	0.3399	**0.0027**
Slc27a1	0.0709	**0.005**	**0.0025**
Ptpmt1	0.1963	**<0.0001**	**0.02**
Lpin1	0.2744	0.2161	**0.0041**
Hadh	0.4398	**<0.0001**	0.071
Plaat3	0.3439	**<0.0001**	0.0722
Gpx4	0.3493	0.2151	**0.0123**
Lpcat3	0.086	**0.0065**	**0.0052**
Cyp4f18	0.975	0.1465	**0.0016**
Gde1	0.6162	**0.0159**	**0.023**
Agpat2	0.2703	0.4858	**0.0016**
Echs1	0.5041	**0.0007**	**0.0475**
Acadl	0.8227	**0.0133**	**0.0352**
Pla2g12a	0.2993	**<0.0001**	0.2304
Acacb	0.3744	0.561	**0.0005**

Table 1. Two-way ANOVA results for lipid metabolism-related DEGs. Two-way ANOVA *p* value results for interaction, sex and genotype for data presented in Figure 4d. Statistically significant *p* values ($p < 0.05$) are highlighted in bold.

Uncropped blots

Figure 2e - Representative lanes: 9-12

Original

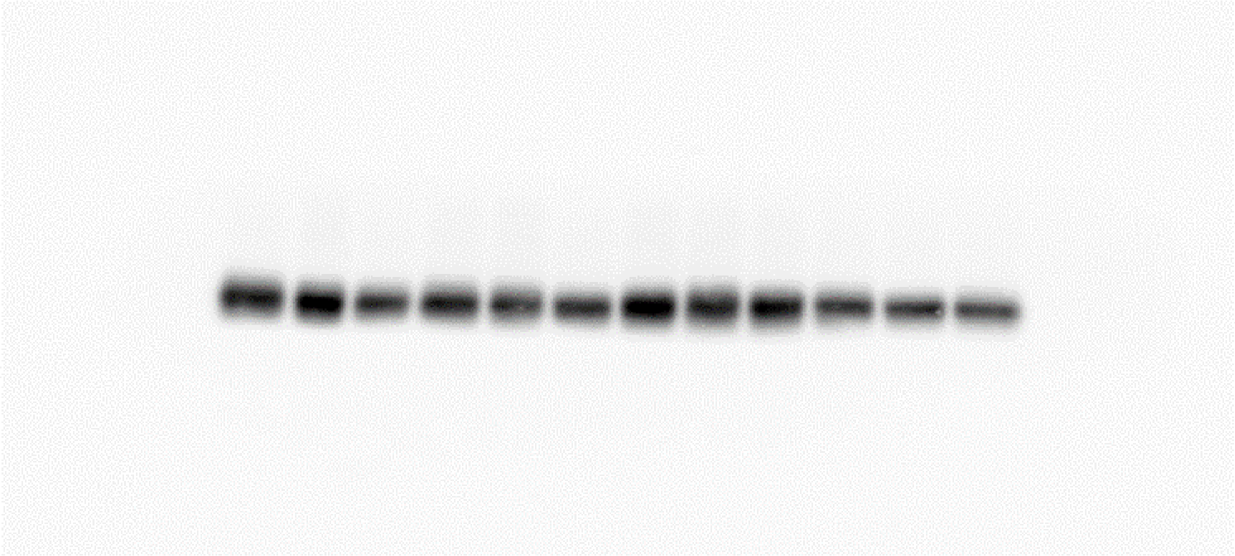

Overlap with marker & labelling

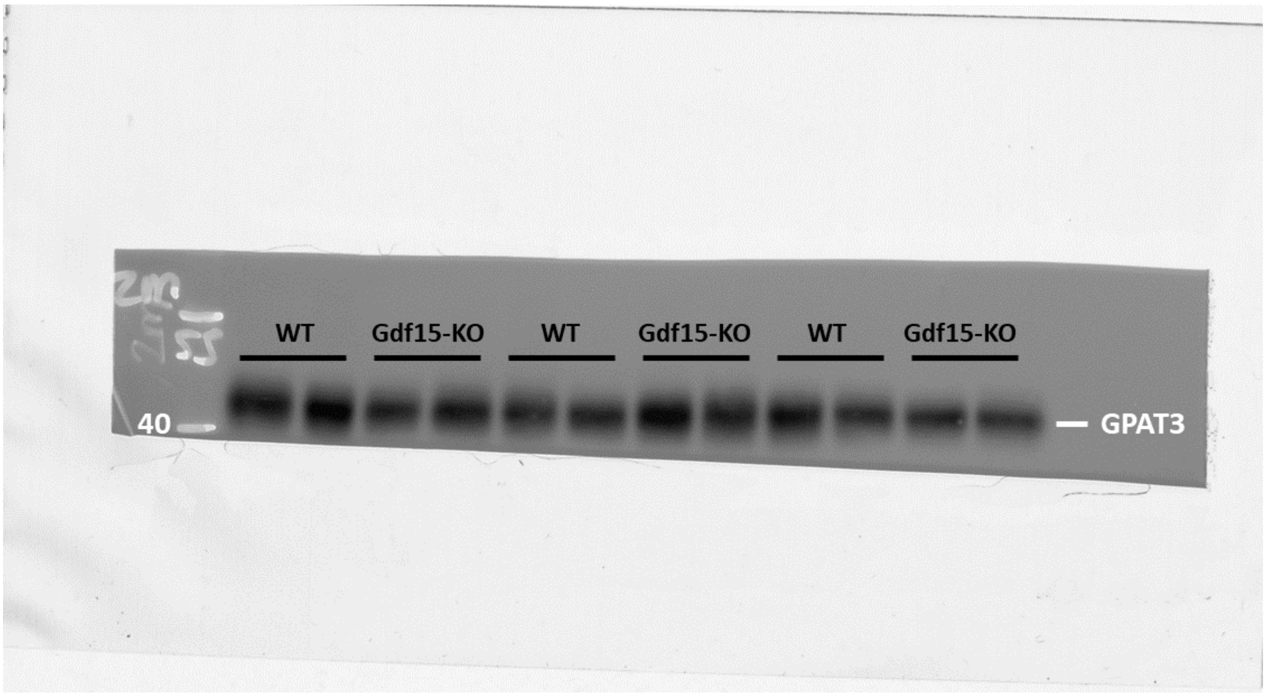

Figure 2h –Representative lanes: 9-12

Original

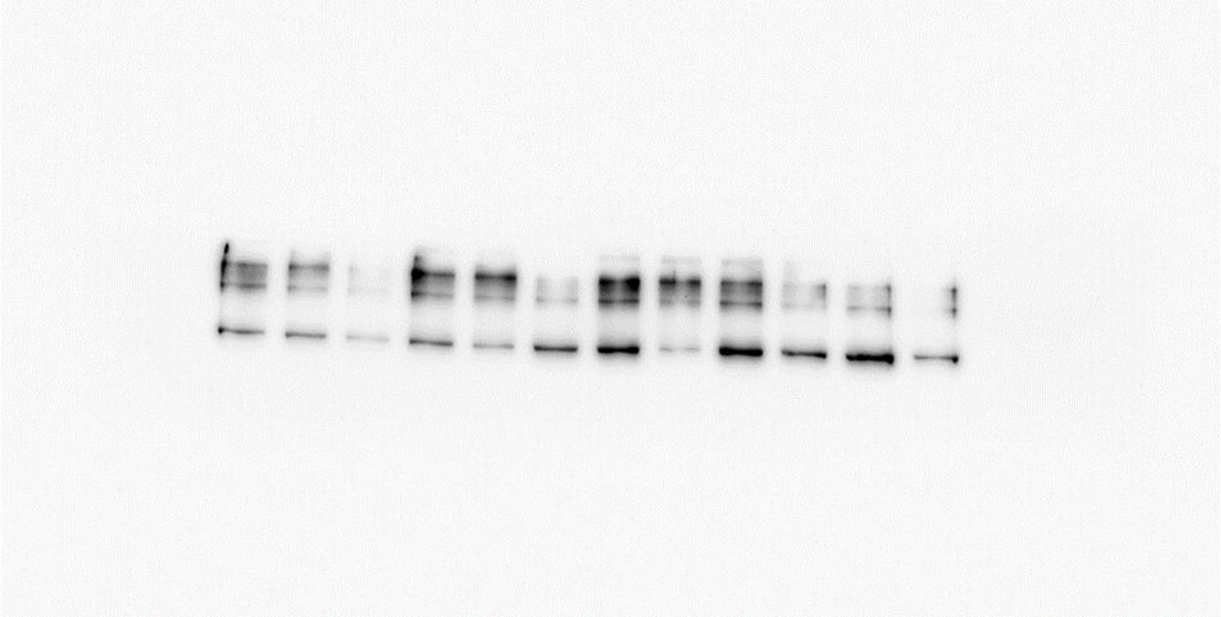

Overlap with marker & labelling

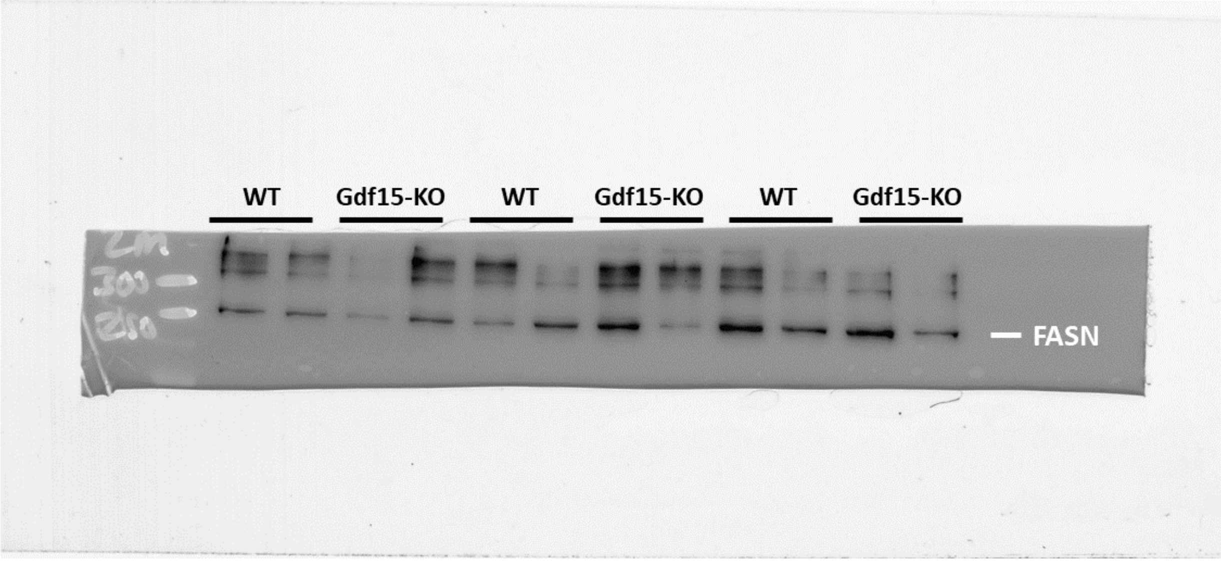

Figure 2n –Representative lanes: 1-4

Original

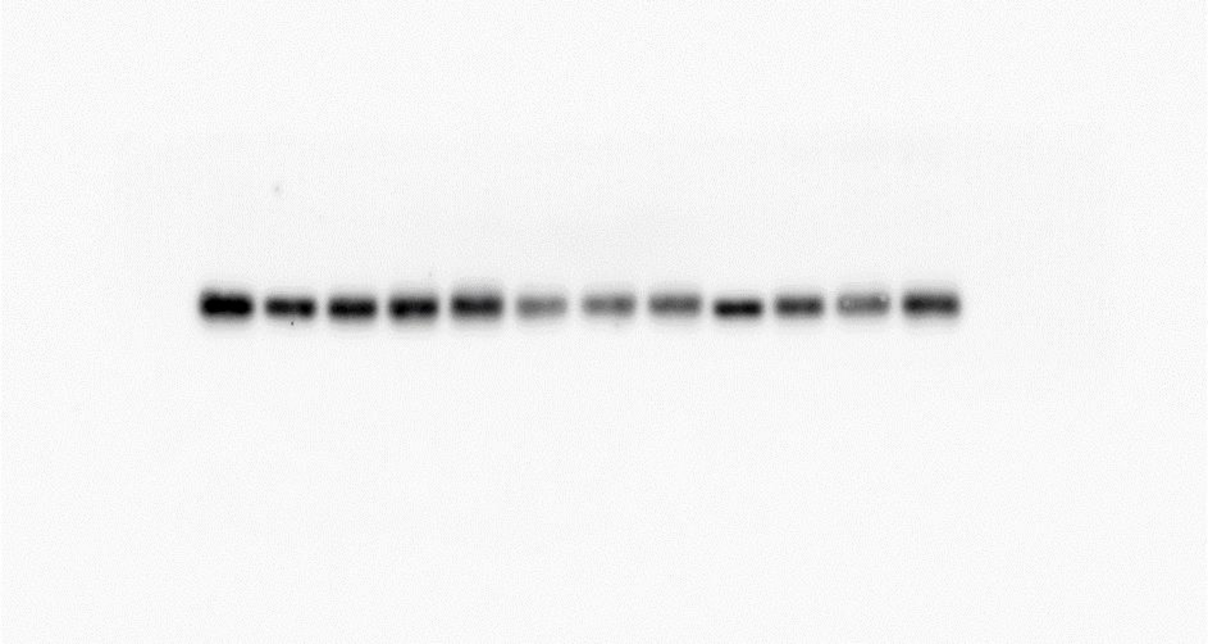

Overlap with marker & labelling

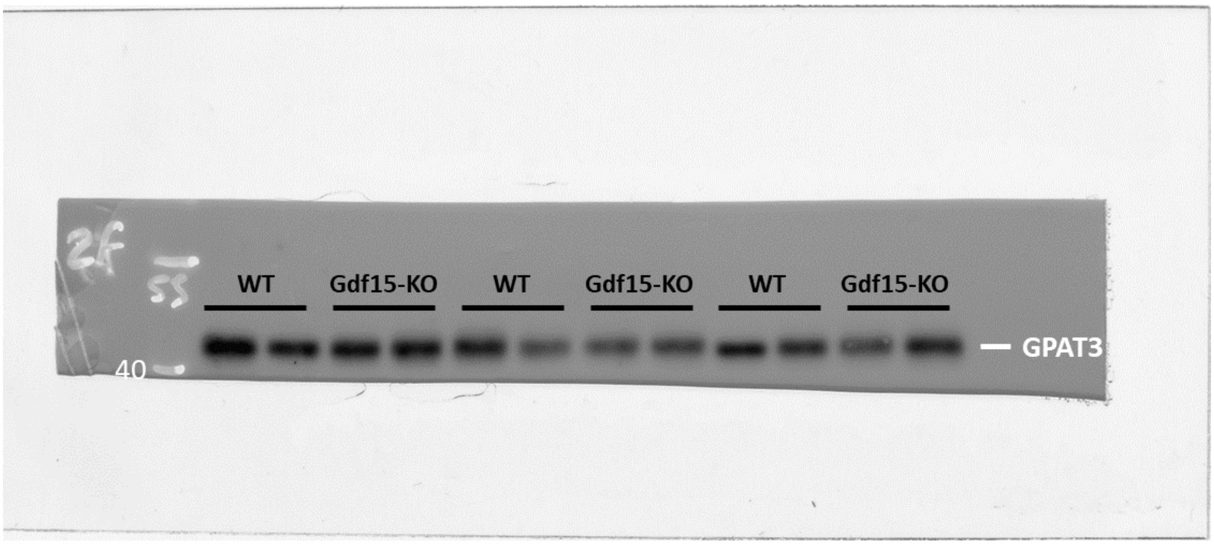

Figure 2q –Representative lanes: 5-8

Original

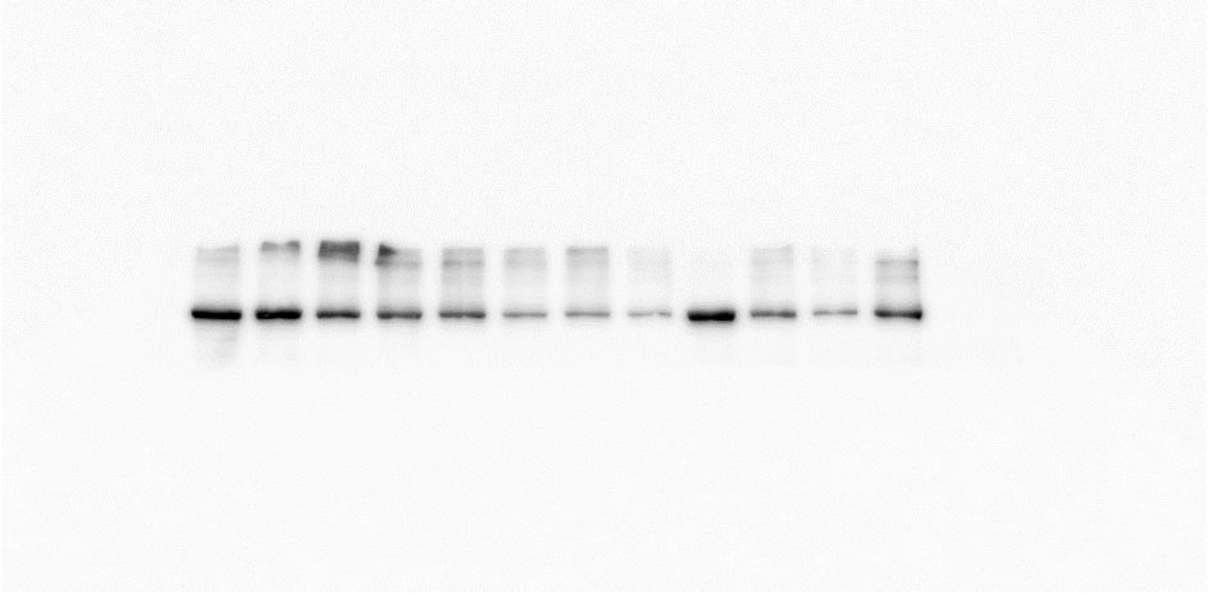

Overlap with marker & labelling

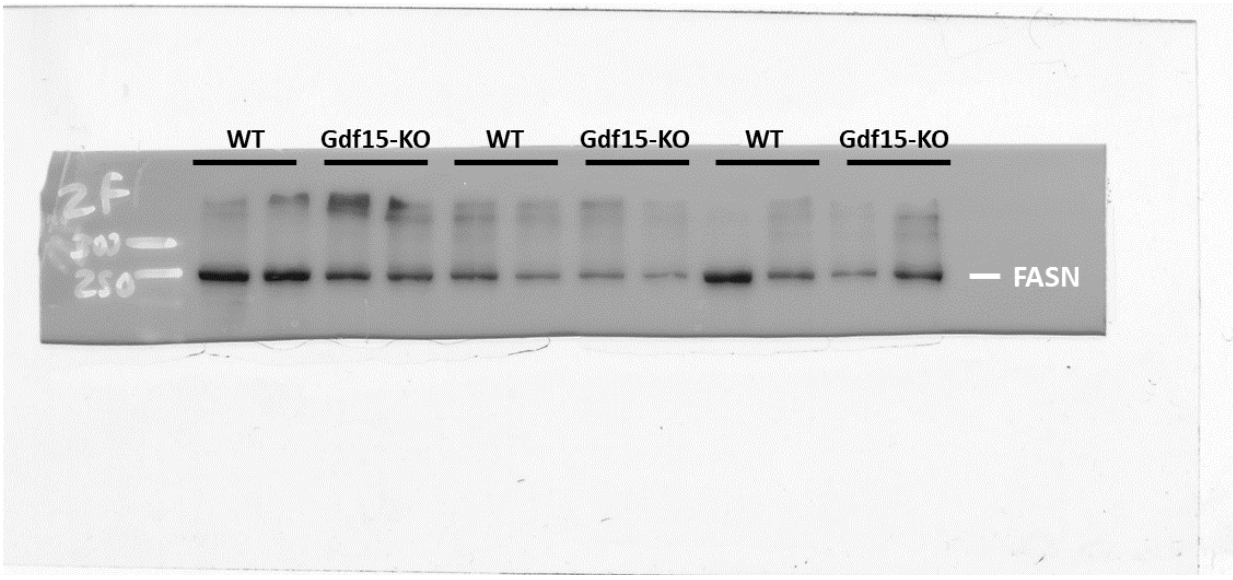

Figure 3e - Representative lanes: 1-4

Original

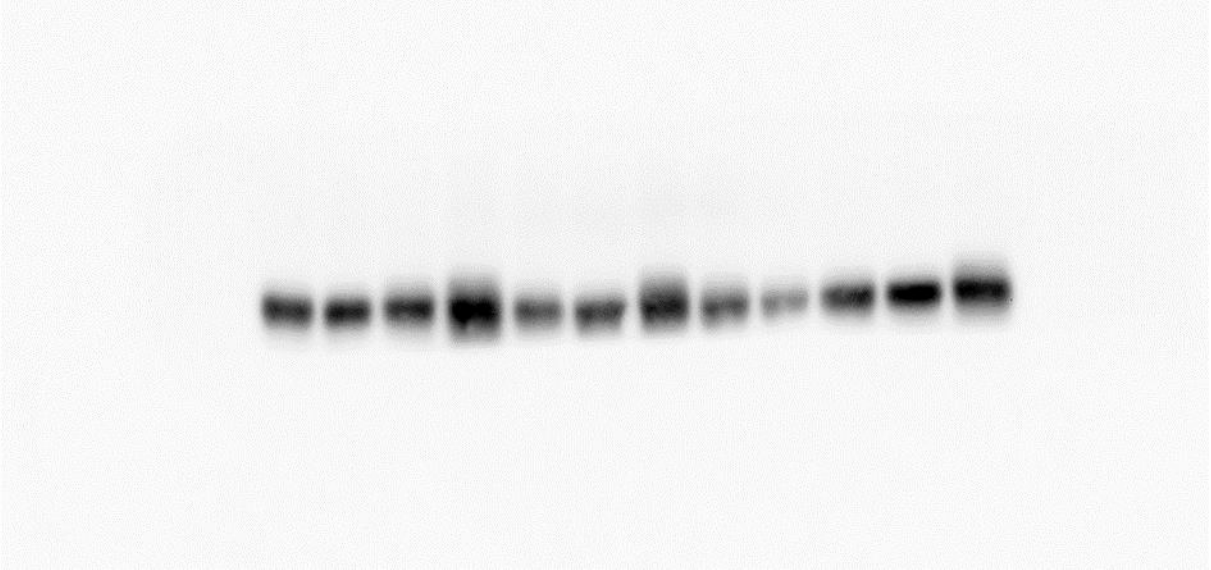

Overlap with marker & labelling

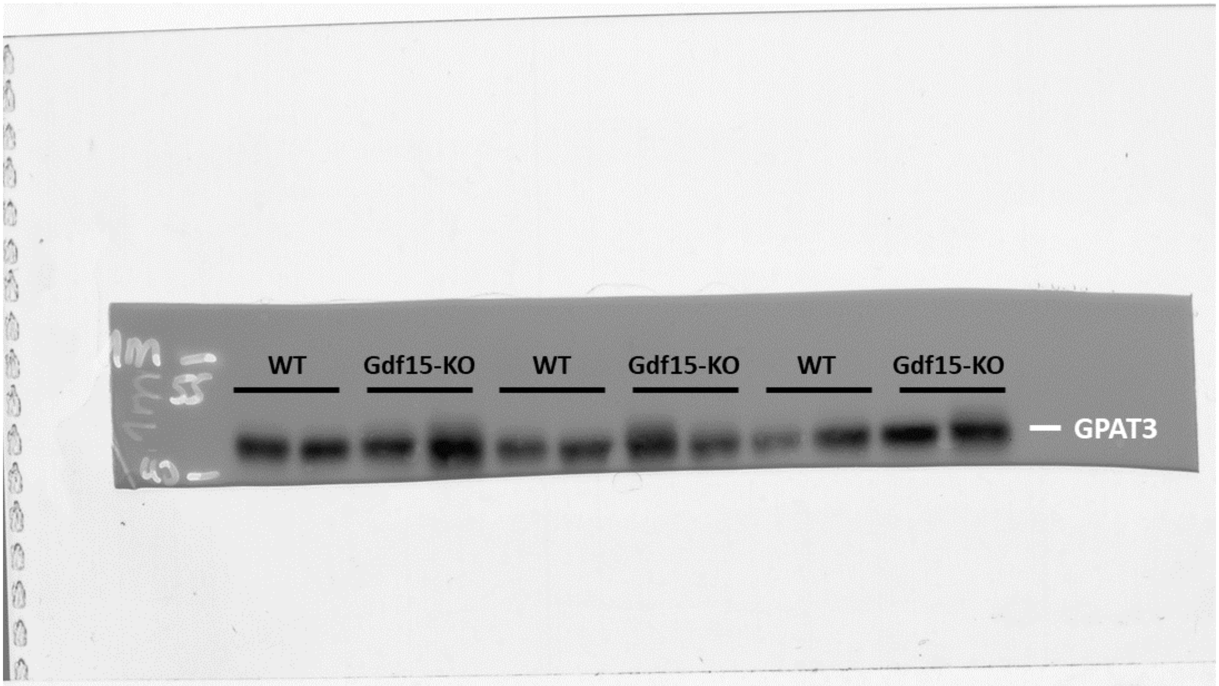

Figure 3h –Representative lanes: 1-4

Original

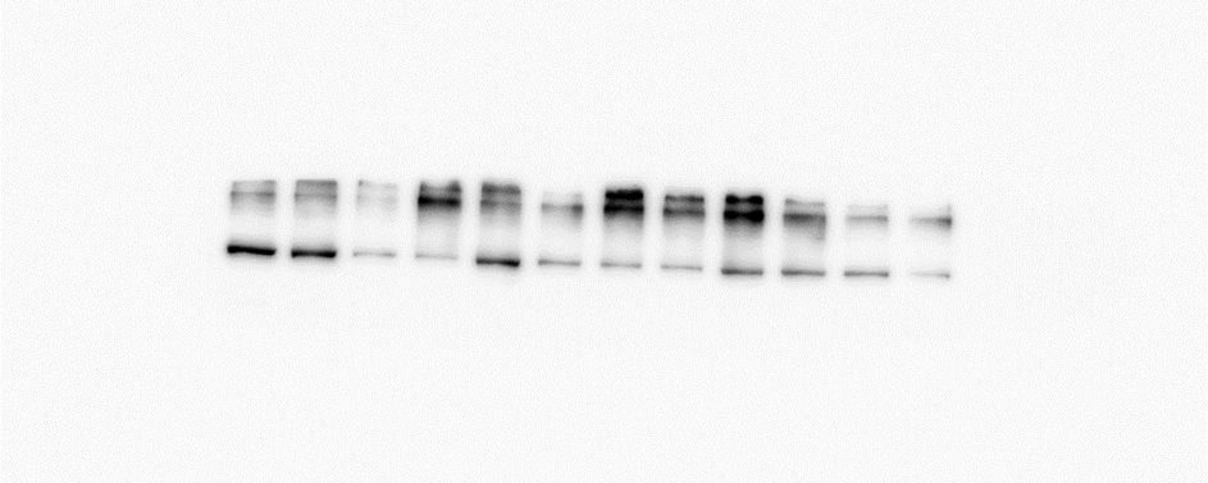

Overlap with marker & labelling

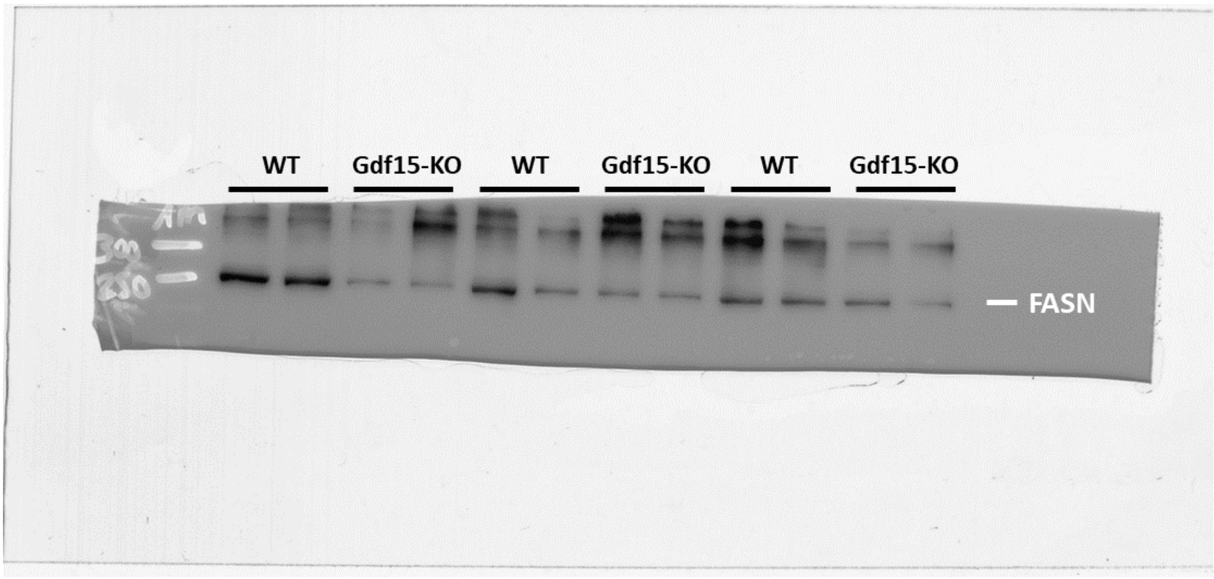

Figure 3n – Representative lanes: 1-4

Original

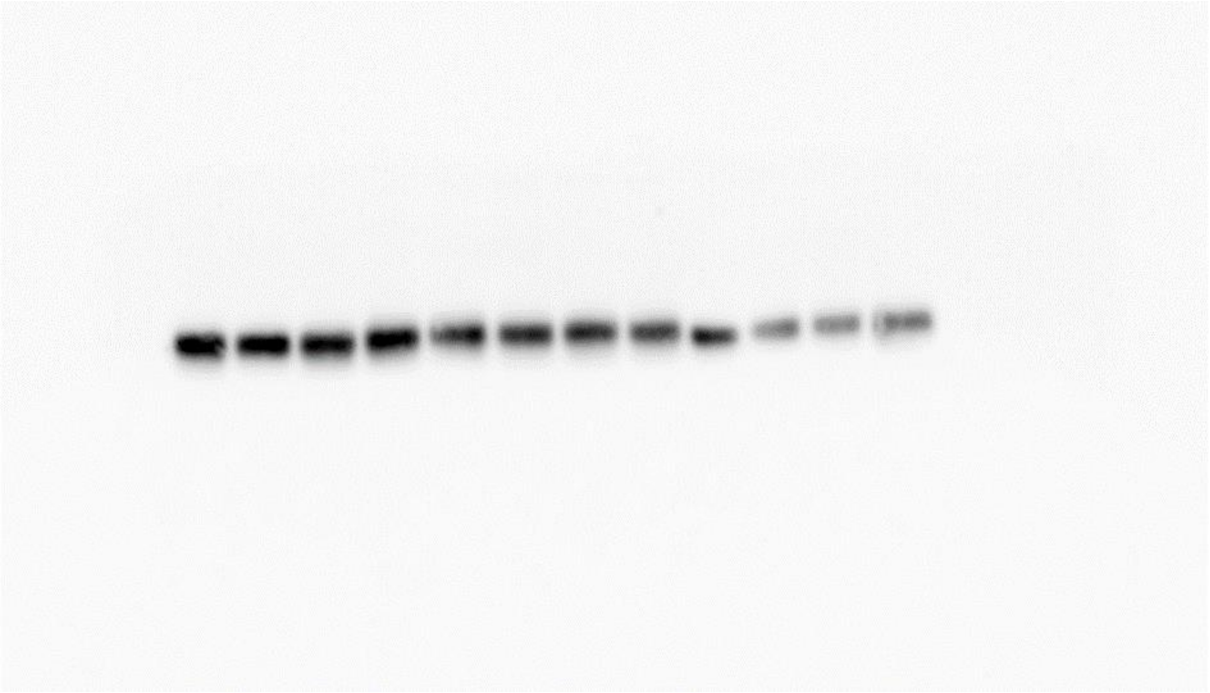

Overlap with marker & labelling

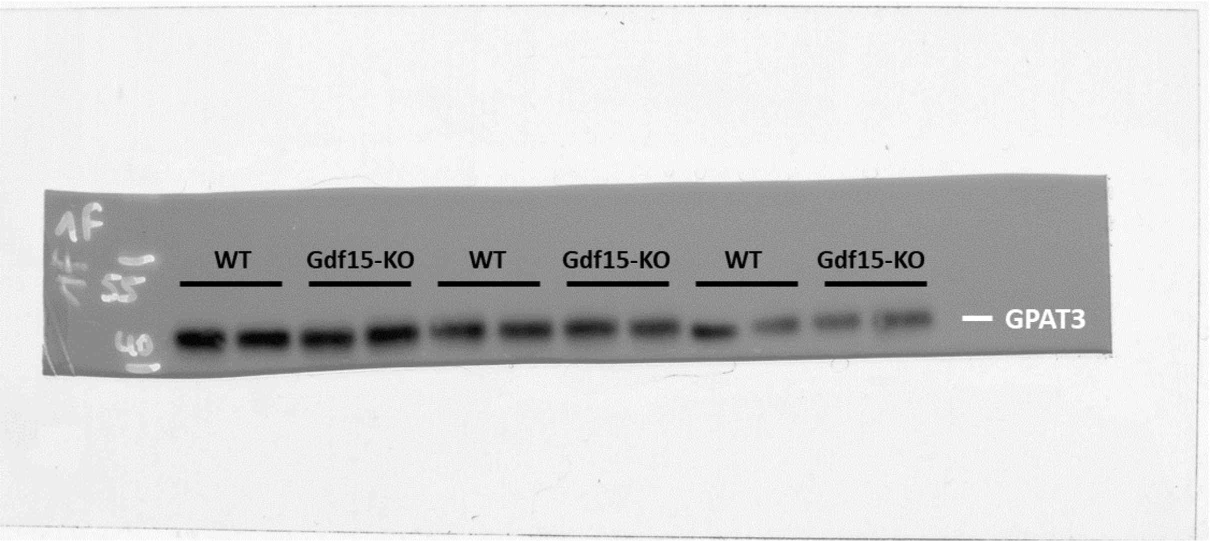

Figure 3q – Representative lanes: 9-12

Original

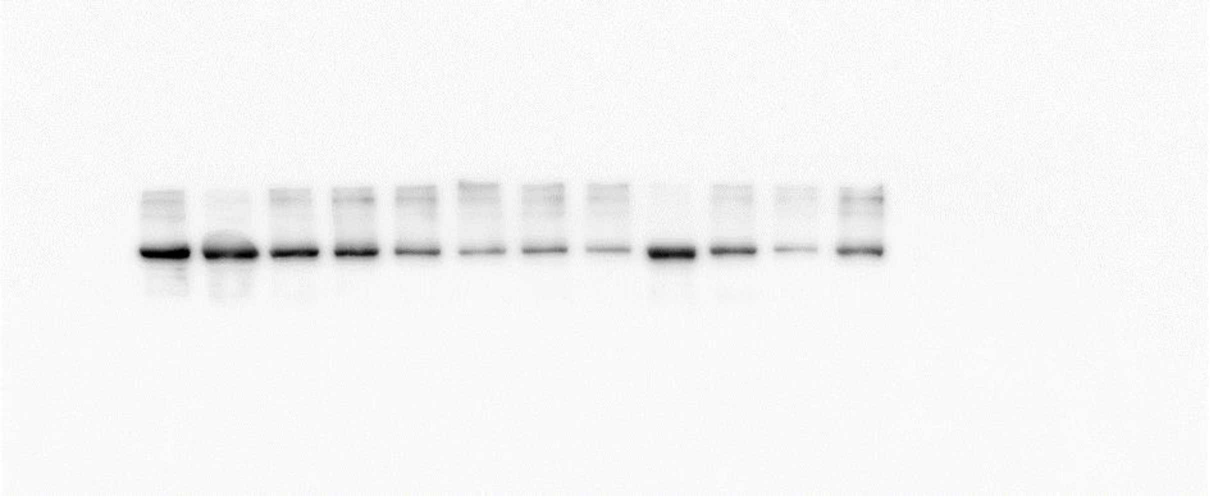

Overlap with marker & labelling

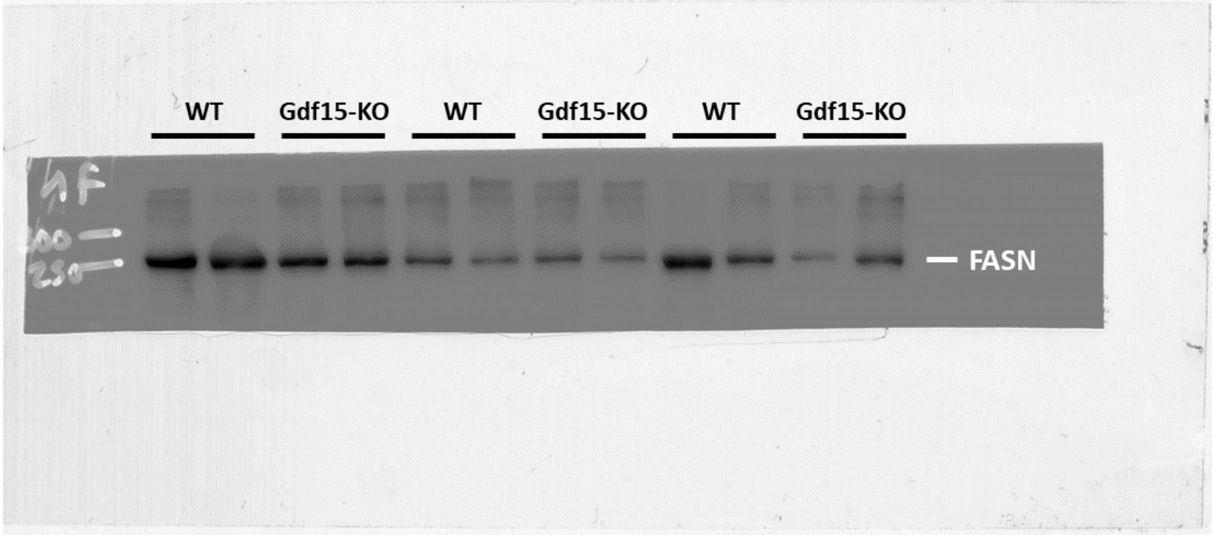

Figure 5g (GPAT3) - Representative lanes: 5-8

Original

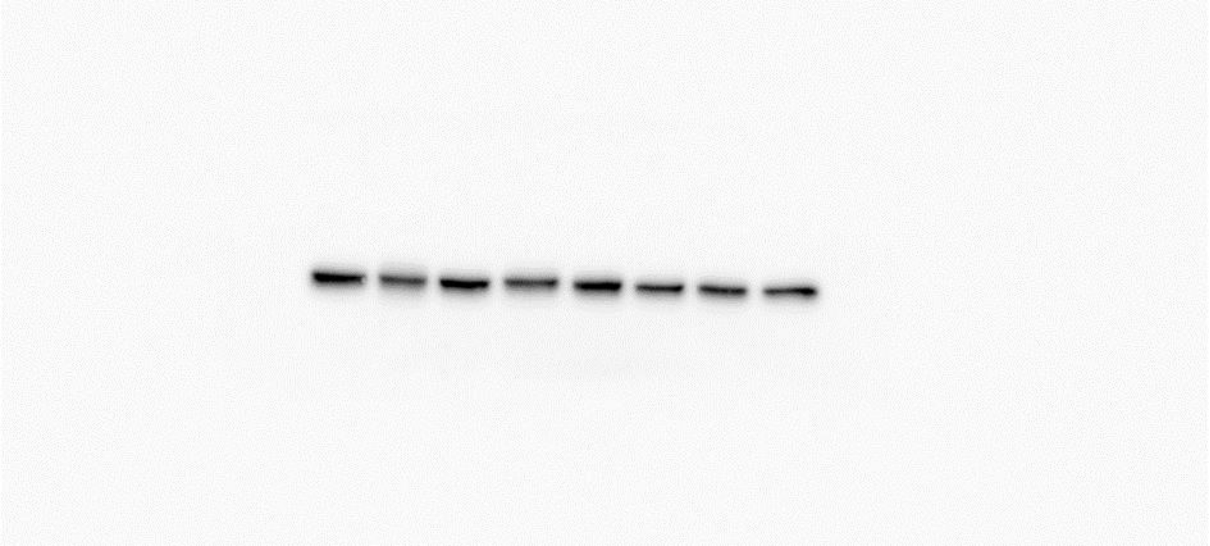

Overlap with marker & labelling

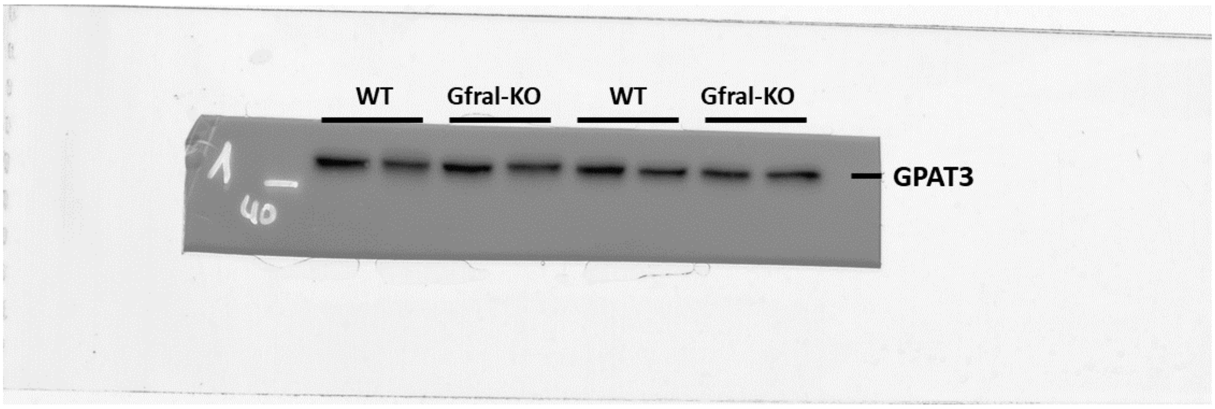

Figure 5g (FASN) – Representative lanes: 5-8

Original

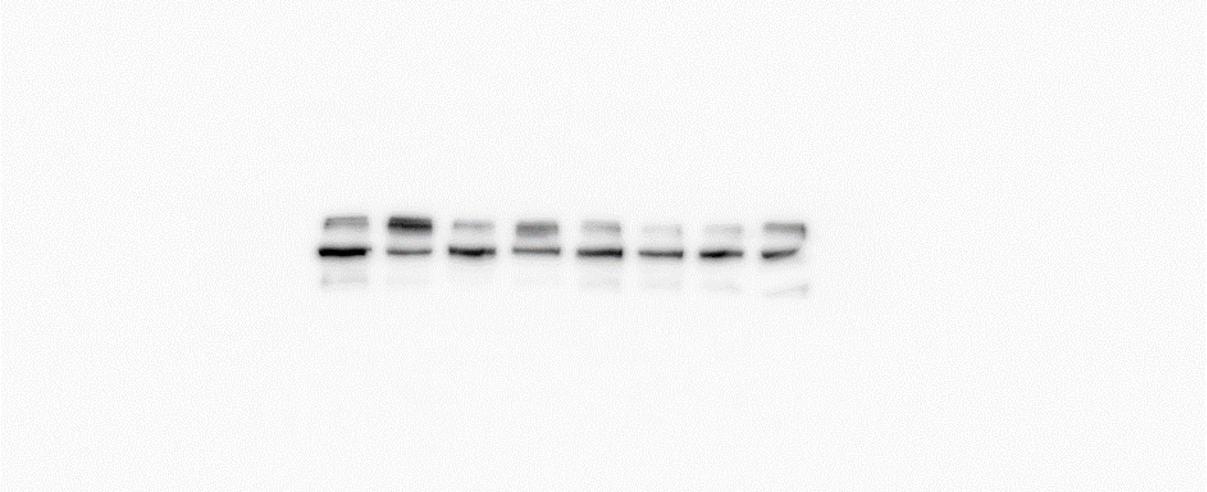

Overlap with marker & labelling

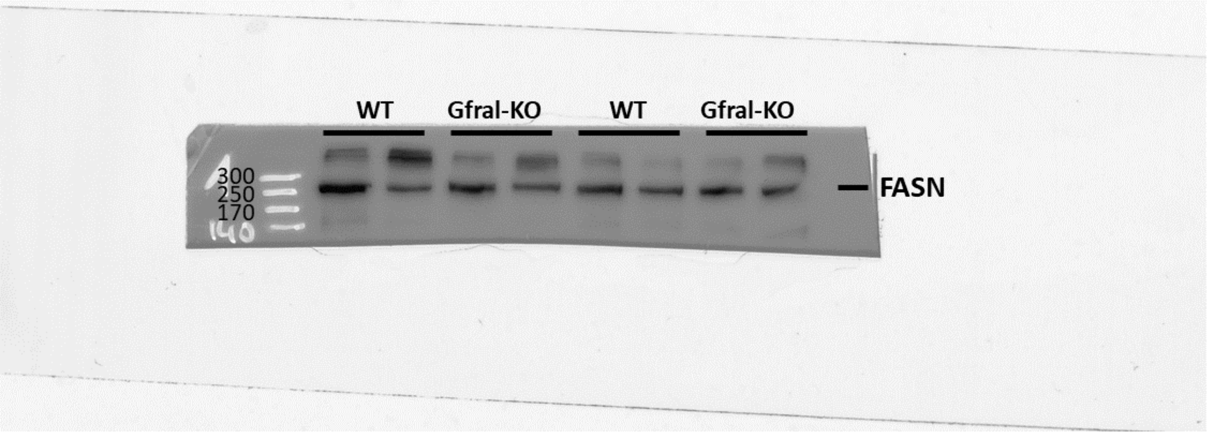

Figure 5p (GPAT3) – Representative lanes: 5-8

Original

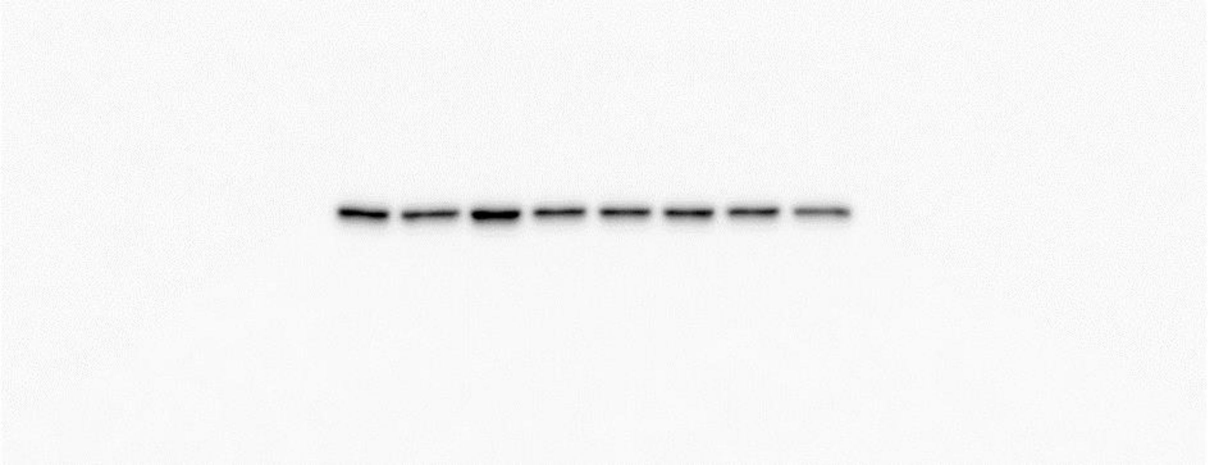

Overlap with marker & labelling

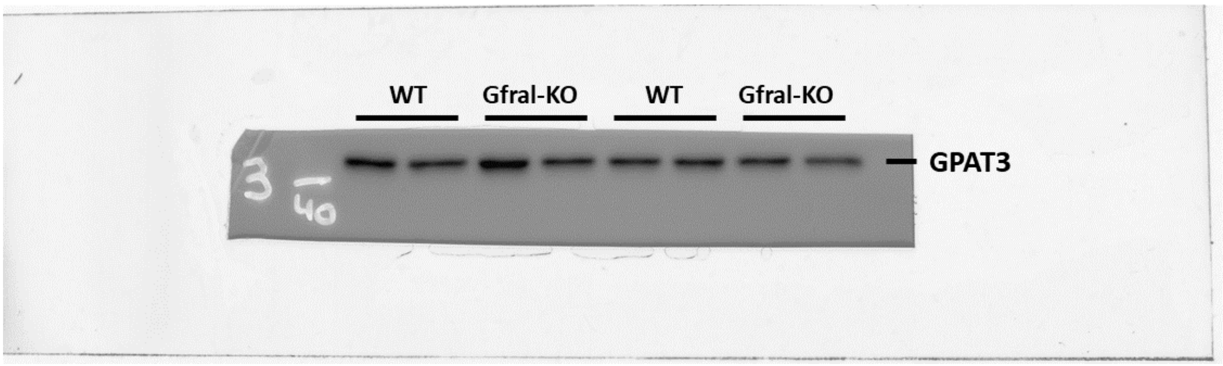

Figure 5p (FASN) - Representative lanes: 5-8

Original

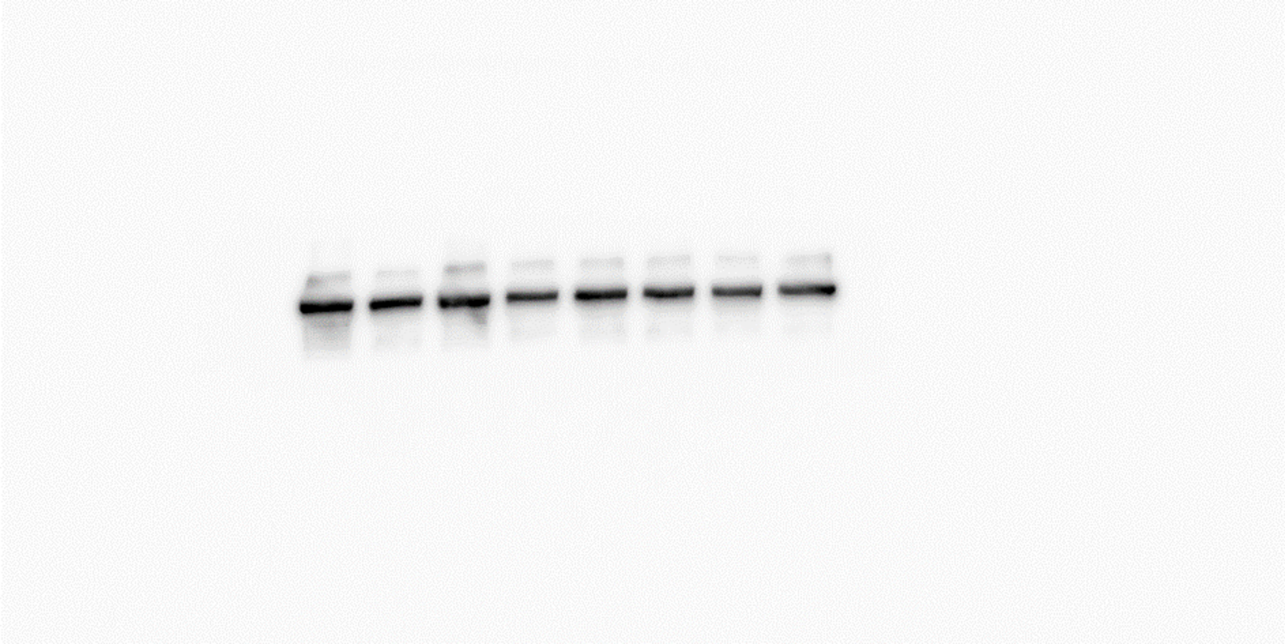

Overlap with marker & labelling

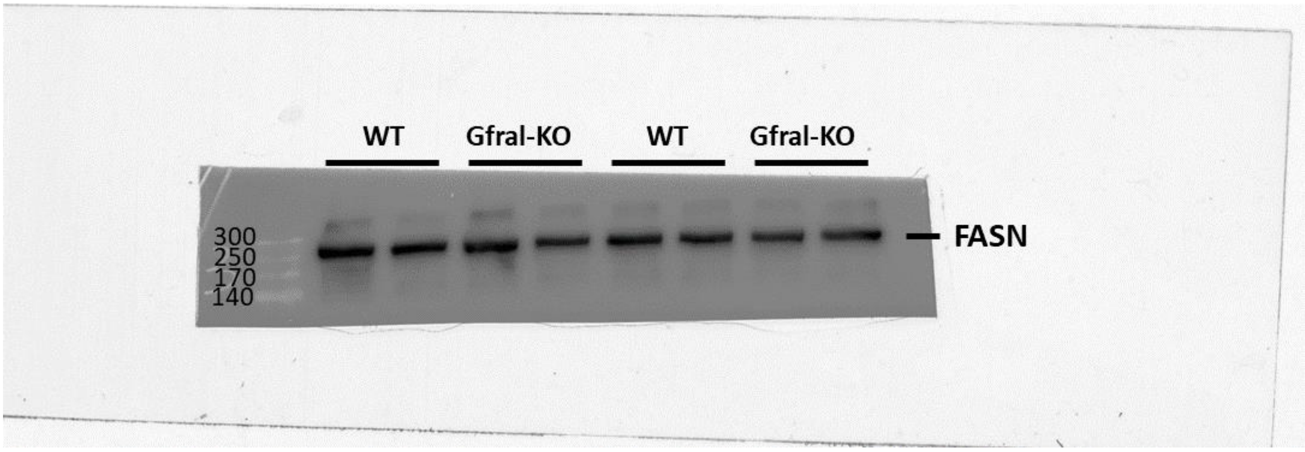

Ponceau staining

Figure 2e, 2h

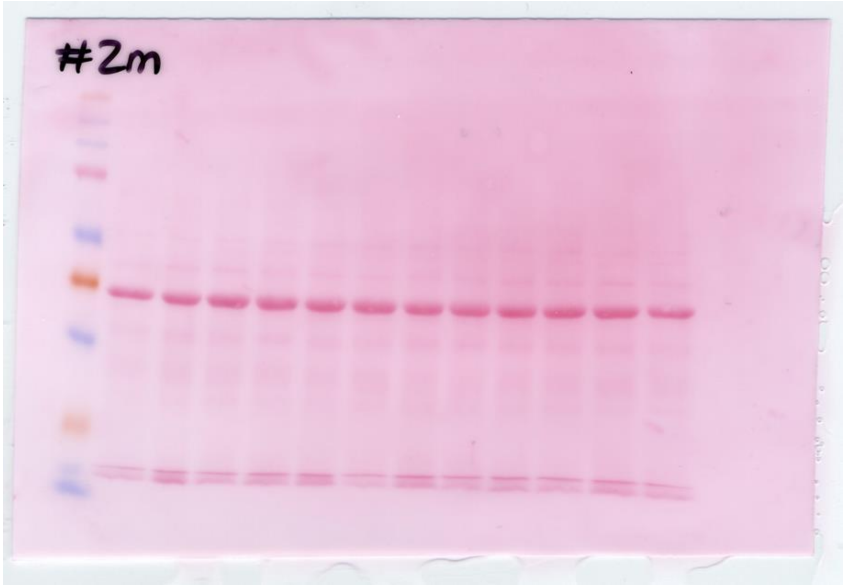

Figure 2n, 2q

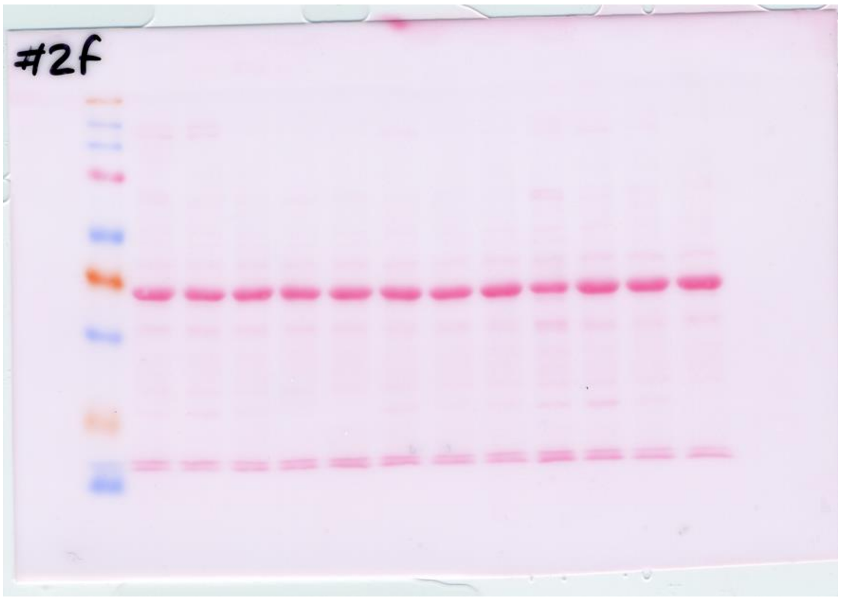

Figure 3e, 3h

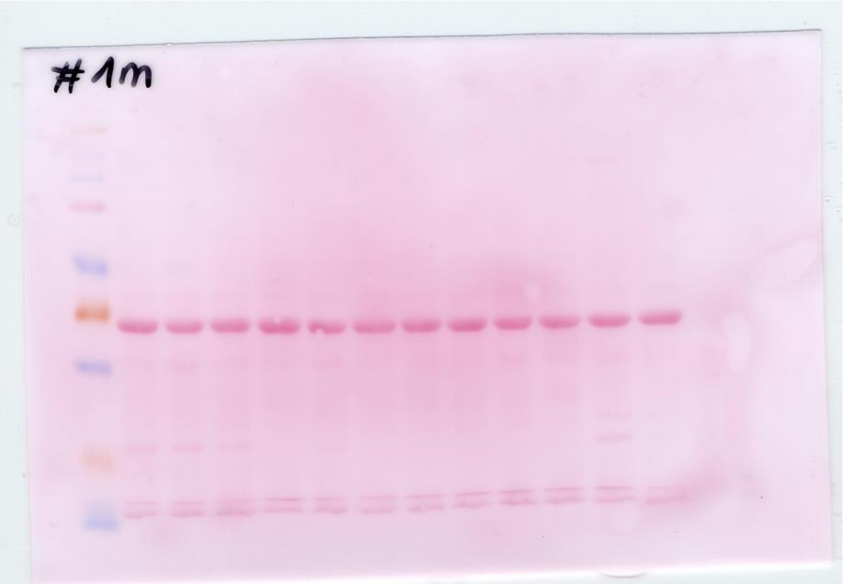

Figure 3n, 3q

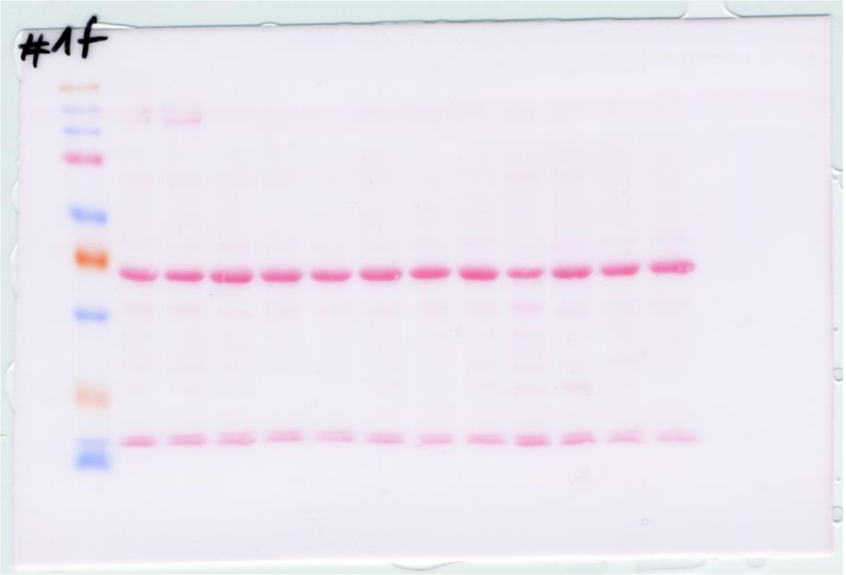

Figure 5g

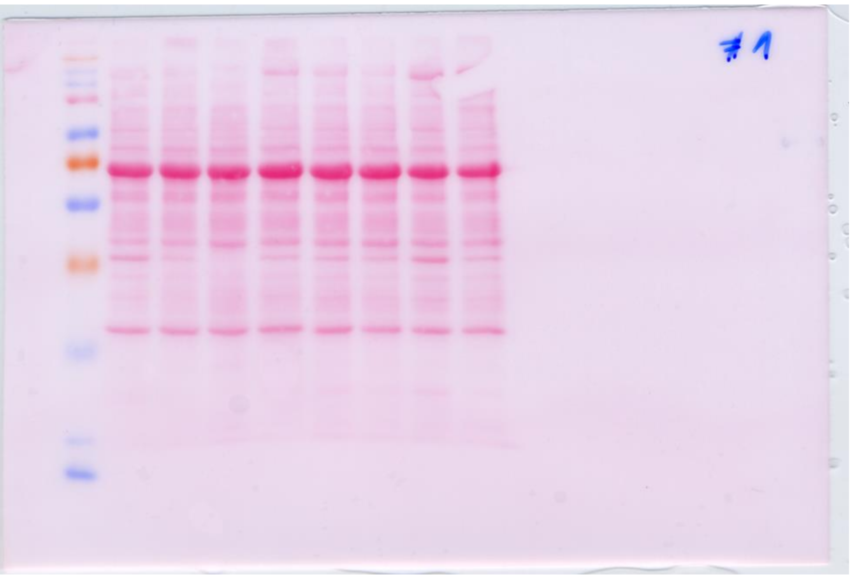

Figure 5p

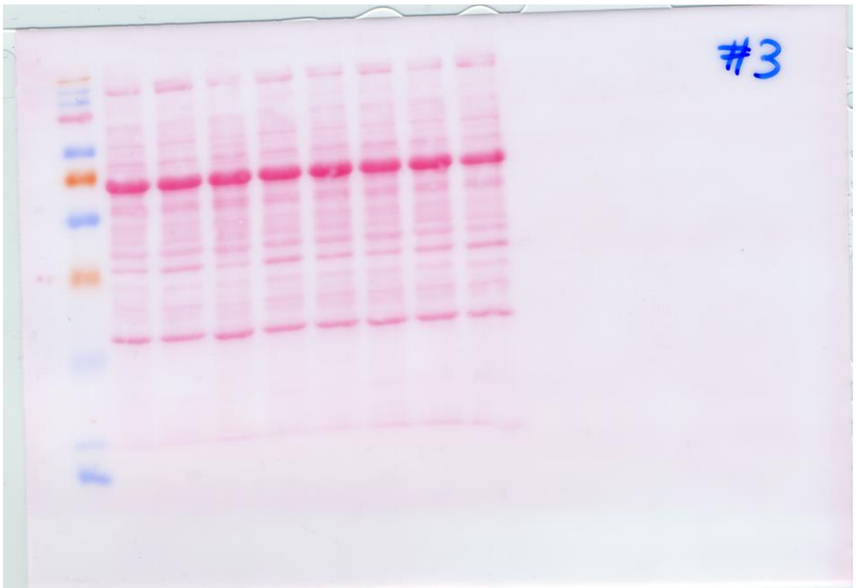

Supplement: Supplementary file 1 — Supplementary Material 1 [file 41598_2024_77448_MOESM1_ESM.pdf]
